# DNA-Binding and Anticancer Activity of Binuclear Gold(I) Alkynyl Complexes with a Phenanthrenyl Bridging Ligand

**DOI:** 10.3390/molecules25051033

**Published:** 2020-02-25

**Authors:** Mona S. Alsaeedi, Bandar A. Babgi, Mostafa A. Hussien, Magda H. Abdellattif, Mark G. Humphrey

**Affiliations:** 1 Department of Chemistry, Faculty of Science, King Abdulaziz University, P.O. Box 80203 Jeddah 21589, Saudi Arabia; m.s.alsaeedi@outlook.com (M.S.A.); mostafa_aly2@yahoo.com (M.A.H.); 2 Department of Chemistry, Faculty of Science, Taif University, Al-Haweiah, P.O. Box 888, Taif 21974, Saudi Arabia; magdah11uk@hotmail.com; 3 Department of Chemistry, College of Science and Arts, King Abdulaziz University, P.O. Box 344 Rabigh 21911, Saudi Arabia; 4 Research School of Chemistry, Australian National University, Canberra, ACT 2601, Australia

**Keywords:** gold(I) alkynyls, phenanthrene, DNA-binding, anticancer activity

## Abstract

3,6-Diethynyl-9,10-diethoxyphenanthrene (**4**) was synthesized from phenanthrene and employed in the synthesis of the binuclear gold(I) alkynyl complexes (R_3_P)Au(C≡C–3-[C_14_H_6_-9,10-diethoxy]-6–C≡C)Au(PR_3_) (R = Ph (**5a**), Cy (**5b**)). The diyne **4** and complexes **5a** and **5b** were characterized by NMR spectroscopy, mass spectrometry, and elemental analysis. UV-Vis spectroscopy studies of the metal complexes and precursor diyne show strong π → π* transitions in the near UV region that red shift by ca. 50 nm upon coordination at the gold centers. The emission spectrum of **4** shows an intense fluorescence band centered at 420 nm which red shifts, slightly upon coordination of **4** to gold. Binding studies of **4**, **5a**, and **5b** against calf thymus DNA were carried out, revealing that **4, 5a**, and **5b** have ≥40% stronger binding affinities than the commonly used intercalating agent ethidium bromide. The molecular docking scores of **4**, **5a**, and **5b** with B-DNA suggest a similar trend in behavior to that observed in the DNA-binding study. Unlike the ligand **4**, promising anticancer properties for **5a** and **5b** were observed against several cell lines; the DNA binding capability of the precursor alkyne was maintained, and its anticancer efficacy enhanced by the gold centers. Such phenanthrenyl complexes could be promising candidates in certain biological applications because the two components (phenanthrenyl bridge and metal centers) can be altered independently to improve the targeting of the complex, as well as the biological and physicochemical properties.

## 1. Introduction

Pioneering studies in supramolecular chemistry by Cram, Lehn, and Pedersen directed attention to “host-guest” systems [1,2,3,4,5]. One particularly interesting application of this concept is the design of new drugs displaying interactions (covalent or noncovalent) with host biological systems such as DNA, enzymes, and proteins [6]. Non-classical (noncovalent) DNA binding modes such as intercalation or groove-binding play a vital role in the pursuit of more efficient and more target-specific drugs displaying fewer side effects [7]. In the field of bioinorganic chemistry, the DNA-binding of platinum [8], ruthenium [9,10], copper [11,12], palladium [13], and gold [14] complexes has been extensively studied due to the array of readily available ligands for coordination, together with the different geometries, coordination numbers, redox potentials, kinetic and thermodynamic characteristics of the resultant complexes. Ligands such as the π-delocalized planar polypyridyls (e.g., diphenylphenazine) have afforded moderate-to-strong DNA-intercalating complexes [15,16]. Modified acetylacetonato [17], amine [18,19], arene [20,21,22], and Schiff base [23,24] ligands have also been used in DNA-binding complex construction. In the pursuit of efficient DNA binders, one well-known strategy is to use a polycyclic aromatic fragment in the ligand that has been designed to form π–π interactions with specific units in the DNA [25,26]. One example employing this approach is the rhodium complex [Rh(trien-Me_2_)(phenanthrene-9,10-diimine)]^3+^ (trien-Me_2_ = 2*R*,9*R*-diamino-4,7-diazadecane) which was specifically designed to intercalate into the 5′-TGCA-3′ sequences in the major groove of DNA; in addition to the water-mediated hydrogen bonds of the trien-Me_2_ ligand, the phenanthrene contributes to the DNA binding through π-stacking forces [27]. More recently, attention has been paid to the use of these complexes as chemotherapeutic agents for cancer [28], DNA conformation probes [29], and DNA cleavage agents [30]. Cisplatin is one of the most important chemotherapeutic agents for the treatment of certain types of cancers. Its mechanism of action is believed to involve interference with DNA replication, interference with transcription, and modification of chromatin [31,32,33]. However, in addition to its solubility problems, the non-target-specific action of the drug and its inactivation in biological environments by reducing agents such as metallothioneins, cisplatin causes many side-effects which limit the doses that can be employed [32,34,35]. As a result of these well-documented problems, extensive research is ongoing to develop new classes of metal complexes that can interfere with cancer cell machinery through interactions with DNA, and this has resulted in an increased interest in developing gold-based anticancer drugs. In this context, gold(I) complexes of the type Au(C≡CR’)(PR_3_) have been reported to exhibit very strong inhibition of the enzyme thioredoxin reductase (TrxR) and show high antiproliferative activity in tumor cells [36,37,38]. The R and R’ groups in the gold(I) complexes play a significant role in the lipophilicity, stability, and binding affinity and, hence, their mechanism of action [39,40]. Gold(I) alkynyls with P(NMe_2_)_3_ ligands have been reported to have a higher anticancer activity against Caco-2 cells than cisplatin; the complexes feature a favorable combination of hydrophilicity and lipophilicity and good stability under physiological conditions [41]. A range of gold phosphine complexes with alkynyl ligands end-functionalized with flavone-derived moieties have shown remarkable cytotoxicity. They function through two mechanisms, triggering apoptotic cell death via the intrinsic pathway and altering cell cycle progression [42]. 

In the current work, a phenanthrene-based ligand functionalized with terminal alkynyl groups has been constructed, with the goal of obtaining strongly intercalating DNA binders facilitated by the phenanthrene polycyclic aromatic system. In principal, the terminal alkynes can be σ-bonded to a range of metal centers to obtain the corresponding alkynyl complexes; gold(I) phosphine centers were chosen in the present work to generate complexes with potential anticancer activities. The binding constants of the new compounds and complexes were compared to that of the structurally closely related intercalating agent ethidium bromide, and the cytotoxicity of the compounds were assessed against several cancer cell lines and compared to that of cisplatin. 

## 2. Results and Discussion

### 2.1. Synthesis and Characterization

**Scheme 1:**
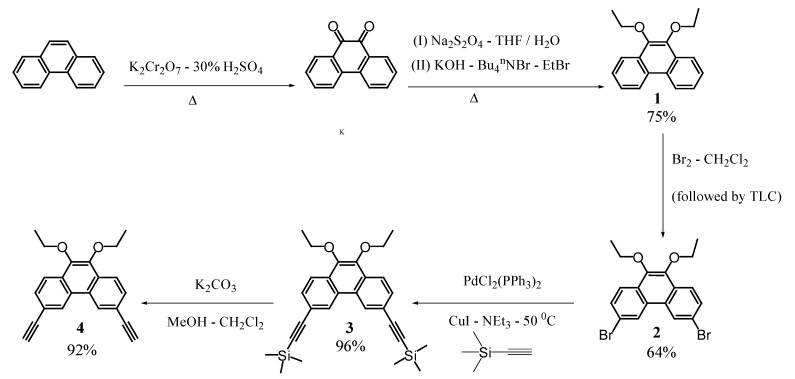
Synthesis of 3,6-diethynyl-9,10-diethoxyphenanthrene (**4**).

**Scheme 2:**
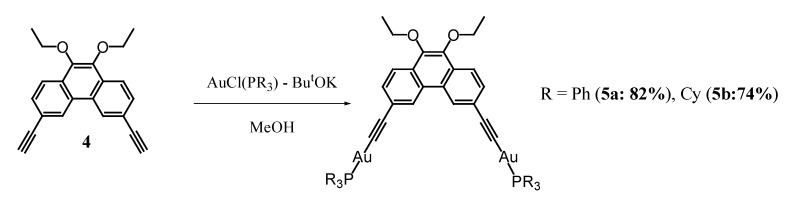
Synthesis of complexes **5a** and **5b**.

The present studies target phenanthrenyl-based alkynyls and their binuclear gold alkynyl complexes. 3,6-Diethynyl-9,10-diethoxyphenanthrene was identified as the key building block. To synthesize this, phenanthrene was subjected to oxidation by a mixture of potassium dichromate and sulfuric acid, leading to the formation of phenanthrene-9,10-dione in a good yield. Then the dione was reduced and, subsequently, treated with ethyl bromide under basic conditions, affording 9,10-diethoxyphenanthrene (**1**). Treating **1** with excess bromine while following the reaction with TLC gave 3,6-dibromo-9,10-diethoxyphenanthrene (**2**) in moderate yields. It is important to highlight that prolonged stirring of the mixture leads to the cleavage of the C-O bond and the formation of 3,6-dibromophenanthrene-9,10-dione (Figures S28 and S29). Compound **2** was coupled with two equivalents of ethynyltrimethylsilane, using PdCl_2_(PPh_3_)_2_ and CuI as catalysts in triethylamine, to give **3**. The silyl-containing compound **3** was desilylated on treatment with K_2_CO_3_, to produce 3,6-diethynyl-9,10-diethoxyphenanthrene (**4**) (Scheme 1). Rather than proceed via bromination, iodination of **1** was tried using several procedures; however, most of these attempts produced 3,6-diiodophenanthrenedione as the main product, together with many inseparable side-products [43,44,45]. The organic precursors were characterized by a combination of elemental analyses, mass spectrometry and IR, and ^1^H and ^13^C–NMR spectroscopies. Finally, the gold(I) alkynyl complexes **5a** and **5b** were obtained successfully by reacting two molar equivalents of the metal-containing precursor with ligand **4**, using modified literature procedures (Scheme 2) [46]. The potassium *tert*-butoxide helps to remove chloride from the gold center, precipitating KCl, and freeing a coordination site for the alkynyl ligand. The formation of the gold alkynyl complexes is a straightforward reaction due to the preference of gold(I) complexes to form linear or trigonal planar complexes (copper(I) and silver(I) have more complicated and less predictable chemistry with acetylenes) [47]. The reaction can be followed easily by ^31^P–NMR because the PR_3_ signal (singlet) is down-field shifted when the chlorido ligand is substituted by the alkynyl ligand [48]. The ^31^P–NMR spectrum of the gold alkynyl complex **5a** shows one singlet around 42 ppm [49], whereas that of **5b** shows a singlet around 56 ppm [50]. IR spectra display bands between 2090 and 2100 cm^−1^ corresponding to the stretching frequencies of the gold-bound C≡C unit. High resolution mass spectra show the molecular ion of the complexes. Other routine analytical techniques were used to verify the identities of the metal complexes, and the resultant data are listed in the experimental section.

### 2.2. Absorption and Emission Spectra

**Figure 1:**
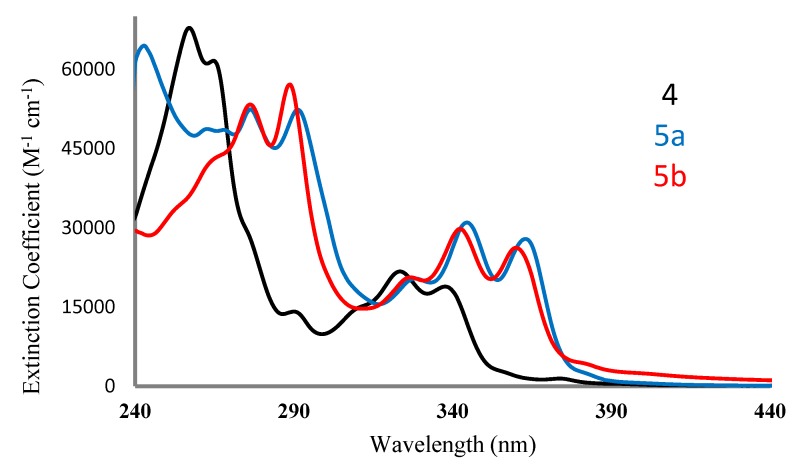
Absorption spectra of **4, 5a**, and **5b** in CHCl_3._

**Figure 2:**
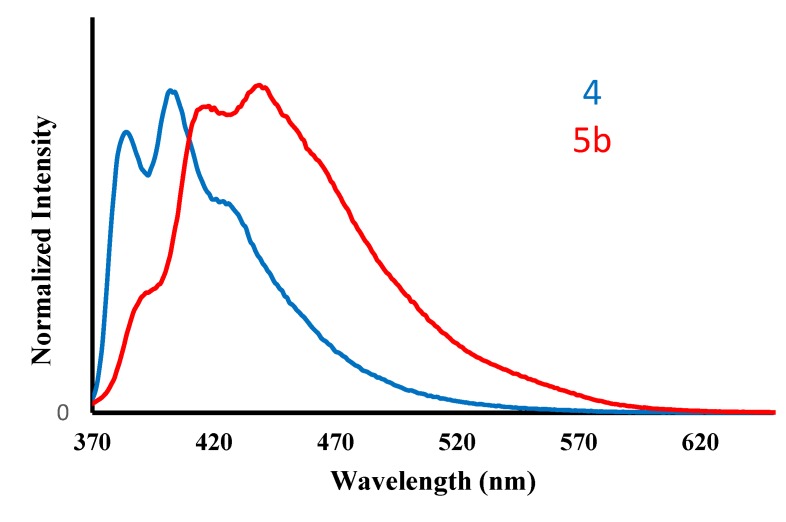
Overlaid normalized emission spectra of **4** and **5b**.

The phenanthrene polycyclic aromatic system is known for its intense absorption bands in the near UV region, as well as its characteristic fluorescence. In this work, the incorporation of gold alkynyl complexes in such a system affords the opportunity to assess the impact of this modification on the photophysical properties. Absorption maxima and intensities obtained from the electronic spectra of **4** and the gold complexes **5a** and **5b** are overlaid in Figure 1. The electronic absorption spectrum of the free ligand **4** displays three bands at 316 nm, 323 nm, and 338 nm. The introduction of Au(PR_3_) fragments at the ligand **4** affords the binuclear gold(I) complexes **5a** and **5b** and leads to a weak red shift in the three absorption bands of the ligand (327 nm, 342 nm, and 362 nm) (Figure 1). The red shifts observed on proceeding to the metal complexes are consistent with a decrease in the energy gap between the HOMO and the LUMO. The free ligand **4** shows an intense broad emission in the near ultraviolet-violet domain upon excitation at 320 nm. Introducing Au(PPh_3_) or Au(PCy_3_) fragments to **4** on proceeding to **5a** and **5b** causes a red shift in the emission bands with almost the same intensities to those of the ligand (both measured at the same concentration) upon excitation at 350 nm (Figure 2).

### 2.3. DNA-Binding Studies

The DNA-binding studies were performed by titrating the compounds with DNA and monitoring the changes spectroscopically. Ethidium bromide was used as a benchmark; it is a fluorophore that binds to DNA by intercalation, and therefore it is used as a fluorescent tag in molecular biology. The binding affinities of the ligand, two gold complexes, and ethidium bromide are summarized in Table 1.

**Figure 3:**
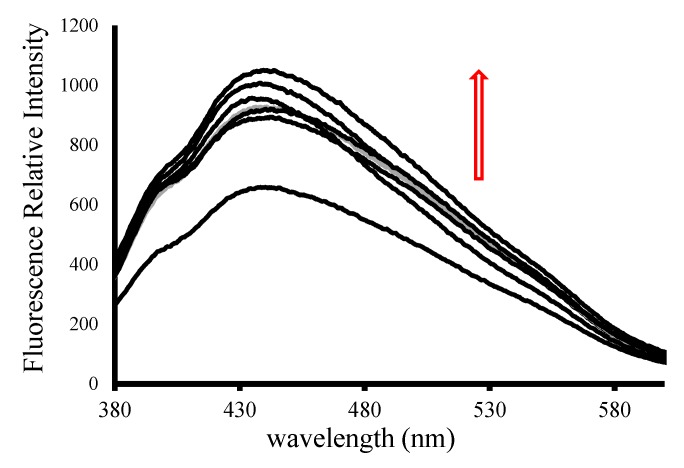
An enhancement in the fluorescence of **5a** upon increasing the concentration of ct-DNA.

The K_b_ values of the compounds show that 3,6-diethynyl-9,10-diethoxyphenanthrene (**4**) has better binding affinity toward ct-DNA than ethidium bromide. Unfavorable steric interactions of the phenyl group of ethidium bromide probably limit its intercalation in DNA, although the amino groups can also contribute via acid–base interactions or hydrogen bonding. In our system, the two flexible ethoxy groups reduce the possibility of steric hindrance as compared with the phenyl group in ethidium bromide, while the acetylene groups can participate in donor–acceptor interactions. Replacing the acidic hydrogen of **4** with gold(I) phosphine metal centers leads to a slight enhancement in the binding constant in the case of the complex with the triphenylphosphine co-ligand, while a slight decrease is observed upon the introduction of the tricyclohexylphosphine co-ligand. PPh_3_ has a smaller cone angle than PCy_3_, leading to better interactions of the phenanthrenyl with the ct-DNA (Table 1). The emission behaviors of **4**, **5a**, and **5b** were monitored as they were titrated with a buffer solution of DNA; the three compounds exhibited a notable enhancement in their emission on increasing the concentration of the ct-DNA, which suggests that these compounds are successfully intercalating, isolating, and rigidifying them, and thereby decreasing deactivation pathways (Figure 3).

### 2.4. Molecular Docking Studies

**Figure 4:**
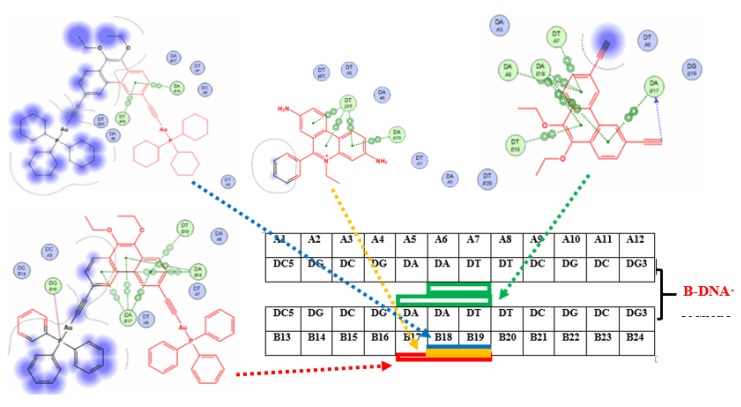
Illustration of the ct-DNA interactions of ethidium bromide, **4**, **5a**, and **5b** and their targeted nucleotides.

Ethidium bromide, the phenanthrene-based ligand **4**, and gold complexes **5a** and **5b** were computationally docked with B-DNA to rationalize the trends from the binding constant values. The data obtained are summarized in Table 2, revealing that all compounds, in this study, target the same location on the B-DNA. In general, the docking scores and pi–pi interactions increased following the order: **5b**, ethidium bromide, **4**, and then **5a**. According to the docking studies, the ethidium bromide establishes pi–pi interactions with deoxyadenosine (DA) and thymidine (DT) nucleotides on strand B (Figure 4 and Table 2). In the case of **4**, it is able to establish interactions with two strands of the ct-DNA, i.e., donor–acceptor interactions are observed on the acetylene with deoxyadenosine (DA) nucleotides, in addition to pi–pi interactions with several deoxyadenosine (DA) and thymidine (DT) nucleotides (Figure 4 and Table 2). In contrast, the gold complexes establish pi–pi interactions with DA and DT on strand B in the major groove (Figure 4 and Table 2). The docking scores follow a similar trend to the K_b_ values. Examining the π-π distances (between the interacting B-DNA on one side and ethidium bromide, **4**, **5a**, and **5b** on the other side), we note that complex **5a** has the shortest distance (3.09 Ǻ), which supports the experimental binding affinity values.

### 2.5. Anticancer Studies

Compounds **3**, **4**, and gold complexes **5a** and **5b** were examined against four cancer cell lines to evaluate their anticancer properties, the results being listed in Table 3. Initial inspection of the results revealed that the organic compounds have no significant anticancer activity while the gold complexes have remarkable cytotoxicity against the four cell lines. The cytotoxicity of cisplatin against MCF-7 and PC-3 cell lines has been used to benchmark the anticancer activity of the gold complexes. We conclude that the gold complexes have comparable or better activities. Several binuclear gold(I) alkynyl compounds have been reported recently with good antitumor activities [36,51], but the drawback to their applications in biological assays is their solubility problems (high lipophilic character). In this regard, the phenanthrene unit in the present system can be altered at Positions 9 and 10, allowing greater control of the lipophilic character of the complexes, and thereby potentially circumventing the solubility concerns.

## 3. Materials and Methods

### 3.1. Materials 

All reactions were carried out using standard Schlenk techniques, under a nitrogen atmosphere. NEt_3_ was distilled over KOH according to standard procedures; other solvents were obtained from Sigma-Aldrich (St. Louis, MO, USA) and were used as received. The term “petrol” refers to a fraction of petroleum ether with a boiling range of 40–60 °C. Experiments containing moisture sensitive compounds were performed using anhydrous solvents and oven-dried (120 °C) glassware. Chromatography was carried out on silica gel 60 particle sizes 0.063 to 0.200 mm (70 to 230 mesh ASTM) or basic ungraded alumina. Copper iodide, *trans*-bis(triphenylphosphine)palladium(II) dichloride, *p*-bromoiodobenzene, tetra-*n*-butylammonium bromide (TBABr), potassium *tert*-butoxide, trimethylsilylacetylene (TMSA), bromine, and bromoethane were purchased commercially and used as received. The following compounds were prepared according to literature procedures: phenanthrene-9,10-dione [52], AuCl(PPh_3_) [53], and AuCl(PCy_3_) [54].

### 3.2. Methods and Instrumentation

**Figure 5:**
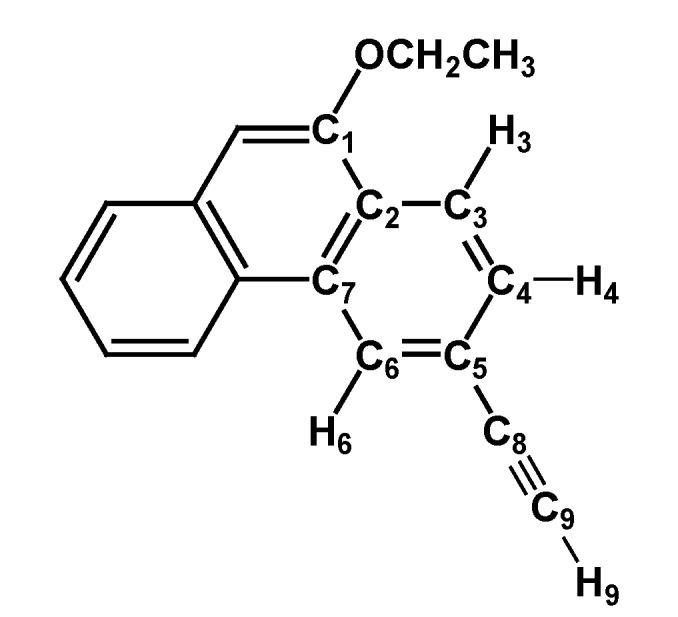
Numbering Scheme for NMR Spectral Assignments.

High-resolution electrospray ionization (ESI) mass spectra were recorded at the Australian National University, using a Bruker Apex 4.7 FTICR-MS instrument (Billerica, MA, USA); all mass spectrometry peaks are reported as *m*/*z* (assignment). Elemental analyses were obtained at King Abdulaziz University. Infrared (IR) spectra were recorded using solid samples on a PerkinElmer Spectrum 100 instrument (Waltham, MA, USA); peaks are reported in cm^−1^. UV-Vis spectra were recorded in 1 cm quartz cells on a MultiSpec-1501 UV-VIS spectrophotometer (Kyoto, Japan) as chloroform solutions; bands are reported in the form wavelength (nm) (extinction coefficient, 10^4^ M^−1^ cm^−1^). UV-Vis emission spectra were recorded for nitrogen-purged chloroform solutions in 1 cm quartz cells using a PerkinElmer LS-55 fluorescence spectrometer; bands are reported in the form wavelength (nm). ^1^H (850 MHz), ^31^P (344 MHz), and ^13^C (214 MHz) NMR spectra were obtained from CDCl_3_ solutions using a Bruker Avance 850 MHz spectrometer. The spectra are referenced to residual chloroform (7.26, ^1^H), CDCl_3_ (77.0, ^13^C), or external H_3_PO_4_ (0.0 ppm, ^31^P); atom labeling follows the numbering in Figure 5. 

### 3.3. Synthesis and Characterization

**Synthesis of 9,10-diethoxyphenanthrene (1).** Tetra(*n*-butyl)ammonium bromide (1.032 g, 3.20 mmol) and Na_2_S_2_O_4_ (6.055 g, 31.81 mmol) were added to a solution of phenanthrene-9,10-dione (2.00 g, 9.60 mmol) in THF (60 mL) and H_2_O (60 mL). After 5 min, KOH (3.563 g, 73.63 mmol) was added to the reaction mixture followed by EtBr (6.388 g, 58.63 mmol). The color of the solution changed to red. After completing the addition, the resulting mixture was stirred at reflux for 5 h. The reaction mixture was cooled to room temperature and the organic phase was extracted with diethyl ether (75 mL). The organic layer was dried over MgSO_4_ and the solvent was removed under reduced pressure. The crude product was purified by column chromatography on silica, eluting with CH_2_Cl_2_/petrol (1:1) to afford **1** (1.485 g, 75%) as a yellow oil. HR ESI MS [C_18_H_18_O_2_]: Calcd. 266.1307, found 266.1302. IR (liquid): 1323, 1107 ν(C-O). UV-Vis (CHCl_3_): 304 [25.31], 341 [5.75], and 358 [4.41]. ^1^H–NMR (CDCl_3_): δ 1.51 (t, ^3^*J_HH_* = 8 Hz) [6H, 2 × CH_3_], 4.31 (q, ^3^*J_HH_* = 8 Hz) [4H, 2 × OCH_2_], 7.60 (m) [4H, H_4_ and H_5_], 8.26 (d, ^3^*J_HH_* = 8 Hz) [2H, H_6_], and 8.64 (d, ^3^*J_HH_* = 8 Hz) [2H, H_3_]. ^13^C–NMR (CDCl_3_): 15.94 (2C, 2 × CH_3_), 69.04 (2C, 2 × OCH_2_), 122.37 (2C, C_5_), 125.67 (2C, C_6_), 126.72 (2C, C_3_), 128.63 (4C, C_2_-C_7_), 129.75 (2C, C_4_), and 143.08 (2C, C_1_).

**Synthesis of 3,6-dibromo-9,10-diethoxyphenanthrene (2).** A mixture of bromine (2.315 g, 14.28 mmol) and CH_2_Cl_2_ (70 mL) was added dropwise to a solution of **1** (1.927 g, 7.14 mmol) in CH_2_Cl_2_ (30 mL) over 1 h at room temperature, and the resultant mixture was stirred for a further 20 min. The reaction mixture was washed with a solution of Na_2_SO_3_ (30 mL, 1.0 M), and the organic phase was collected and dried over MgSO_4_. The solvent was reduced in volume under reduced pressure and the crude product was purified by column chromatography on silica, eluting with CH_2_Cl_2_/petrol (1:1) to afford **2** (1.97 g, 64%) as a yellow powder. HR ESI MS [C_18_H_16_^79^Br_2_O_2_]: Calcd. 421.9517, found 421.9518. HR ESI MS [C_18_H_16_^79^Br^81^BrO_2_]: Calcd. 423.9497, found 423.9498. HR ESI MS [C_18_H_16_^81^Br_2_O_2_]: Calcd. 425.9476, found 425.9478. Elemental analysis for C_18_H_16_Br_2_O_2_, Calcd (found): C, 50.97 (50.52); H, 3.80 (3.48). IR (solid): 1614 cm^−1^ ν(C=C), 1345 ν(C-O), and 813 ν(C-Br). UV-Vis (CHCl_3_): 304 [1.26], 316 [1.29], and 354 [1.69]. ^1^H–NMR (CDCl_3_): δ 1.49 (t, ^3^*J_HH_* = 7 Hz) [6H, 2 × CH_3_], 4.28 (q, ^3^*J_HH_ =* 7 Hz) [4H, 2 × OCH_2_], 7.71 (dd, ^3^*J_HH_* = 9 Hz, ^4^*J_HH_* = 2 Hz) [2H, H_4_], 8.12 (d, ^3^*J_HH_* = 9 Hz) [2H, H_6_], and 8.66 (d, ^4^*J_HH_* = 2 Hz) [2H, H_3_]. ^13^C–NMR (CDCl_3_): 15.87 (2C, 2 × CH_3_), 69.16 (2C, 2 × OCH_2_), 120.37 (2C, C_4_), 124.30 (2C, C_6_), 125.39 (2C, C_3_), 127.42, 128.86 (4C, C_2_-C_7_), 130.50 (2C, C_5_), and 142.89 (2C, C_1_).

**Synthesis of 3,6-bis(trimethylsilylethynyl)-9,10-diethoxyphenanthrene (3).** Trimethylsilylacetylene (0.174 g, 1.77 mmol), *trans*-PdCl_2_(PPh_3_)_2_ (0.041 g, 0.05 mmol) and CuI (0.005 g, 0.02 mmol) were added to a solution of compound **2** (0.376 g, 0.88 mmol) in NEt_3_ (15 mL) under a N_2_ atmosphere, and the mixture was stirred at 50 °C overnight. The solvent was reduced in volume under reduced pressure and the crude product was purified by column chromatography on silica, eluting with CH_2_Cl_2_/petrol (1:1) to afford **3** (0.391 g, 96%) as a yellow solid. HR ESI MS [C_28_H_34_O_2_Si_2_]: Calcd. 458.2097, found 458.2098. Elemental analysis for C_28_H_34_O_2_Si_2_, Calcd (found): C, 73.31 (73.04) and H, 7.47 (7.11). IR (solid): 2157 ν(C≡C). UV-Vis (CHCl_3_): 319 [1.20], 332 [1.84], and 348 [1.69]. ^1^H–NMR (CDCl_3_): δ 0.31 (s, 9H, SiMe_3_), 1.52 (t, ^3^*J_HH_* = 7 Hz) [6H, 2 × CH_3_], 4.30 (q, ^3^*J_HH_* = 7 Hz) [4H, 2 × OCH_2_], 7.66 (dd, ^3^*J_HH_* = 9 Hz, ^4^*J_HH_* = 2 Hz) [2H, H_4_], 8.15 (d, ^3^*J_HH_* = 9 Hz) [2H, H_6_], and 8.73 (d, ^4^*J_HH_* = 2 Hz) [2H, H_3_]. ^13^C–NMR (CDCl_3_): 0.00 (3C, SiMe_3_), 15.84 (2C, 2 × CH_3_), 69.09 (2C, 2 × OCH_2_), 94.83 (2C, C_9_), 105.51 (2C, C_8_), 120.44 (2C, C_5_), 122.31 (2C, C_4_), 126.61 (2C, C_6_), 127.61 (2C, C_3_), 129.60 (2C, C_7_), 129.98 (2C, C_2_), and 143.62 (2C, C_1_).

**Synthesis of 3,6-diethynyl-9,10-diethoxyphenanthrene (4).** Compound **3** (2.649 g, 5.77 mmol) and K_2_CO_3_ (4.791 g, 34.67 mmol) were stirred in a mixture of methanol (20 mL) and dichloromethane (20 mL) under N_2_ at room temperature for 6 h. The solvent was reduced in volume under reduced pressure and the crude product was purified by column chromatography on silica, eluting with CH_2_Cl_2_/petrol (1:1) to afford **4** (1.338 g, 92%) as a yellow solid. HR ESI MS [C_22_H_18_O_2_]: Calcd. 314.1307, found 314.1308. Elemental analysis for C_22_H_18_O_2_, Calcd (found): C, 84.05 (83.96) and H, 5.77 (5.41). IR (solid): 3292 ν(≡C-H) and 2154 ν(C≡C). UV-Vis (CHCl_3_): sh 316 [1.67], 323 [2.17], and 338 [1.88]. ^1^H–NMR (CDCl_3_): δ 1.48 (t, ^3^*J_HH_* = 15 Hz) [6H, 2 × CH_3_], 3.19 (s) [2H, H_9_], 4.30 (q, ^3^*J_HH_ =* 15 Hz) [4H, 2 × OCH_2_], 7.69 (dd, ^3^*J_HH_* = 18 Hz, ^4^*J_HH_* = 3 Hz) [2H, H_4_], 8.19 (d, ^3^*J_HH_* = 18 Hz) [2H, H_6_], and 8.74 (d, ^4^*J_HH_* = 3 Hz) [2H, H_3_]. ^13^C–NMR (CDCl_3_): 15.86 (2C, 2 × CH_3_), 69.18 (2C, 2 × OCH_2_), 77.74 (2C, C_8_), 84.12 (2C, C_9_), 119.57 (2C, C_5_), 122.59 (2C, C_4_), 126.92 (2C, C_6_), 127.64 (2C, C_3_), 129.98 (2C, C_7_), 130.09 (2C, C_2_), and 143.73 (2C, C_1_).

**Synthesis of (Ph_3_P)Au(C≡C–2-[C_14_H_6_-9,10-diethoxy]-7–C≡C)Au(PPh_3_) (5a).** AuCl(PPh_3_) (0.098 g, 0.19 mmol) and Bu*^t^*OK (0.085 g, 0.19 mmol) were added to a solution of compound **4** (0.031 g, 0.099 mmol) in methanol (15 mL) under a N_2_ atmosphere and the mixture was stirred overnight. The solvent was reduced in volume under reduced pressure and the crude product was purified by column chromatography on silica, eluting with CH_2_Cl_2_/petrol (1:1) to afford **5a** (0.099 g, 82%) as a yellow powder. HR ESI MS [C_58_H_47_^197^Au_2_O_2_P_2_]: Calcd. 1231.2382, found 1231.2382. Elemental analysis for C_58_H_47_Au_2_O_2_P_2_, Calcd (found): C, 56.60 (56.27) and H, 3.77 (3.60). IR (solid): 2096 ν(C≡C). UV-Vis (CHCl_3_): sh 328 [2.04], 343 [3.04], and 363 [2.79]. ^1^H–NMR (CDCl_3_): δ 1.48 (t, ^3^*J_HH_* = 8 Hz) [6H, 2 × CH_3_], 4.26 (q, ^3^*J_HH_* = *7* Hz) [4H, 2 × OCH_2_], 7.62-7.46 (m) [30H, 2 × PPh_3_], 7.71 (dd, ^3^*J_HH_* = 8 Hz, ^4^*J_HH_* = 1 Hz) [2H, H_4_], 8.08 (d, ^3^*J_HH_* = 8 Hz) [2H, H_6_], and 8.78 (d, ^4^*J_HH_* = 1 Hz) [2H, H_3_]. ^13^C–NMR (CDCl_3_): 15.95 (2C, 2 × CH_3_), 69.02 (2C, 2 × OCH_2_), 128.01 (2C, C_8_), 129.13, 129.18, 129.22, 129.28 (36C, 2 × PPh_3_), 129.77 (2C, C_5_) 130.04 (2C, C_4_), 131.52 (2C, C_9_) 134.14, 134.21 (4C, C_3_-C_6_), 134.38, 134.44 (4C, C_2_-C_7_), and 143.29 (2C, C_1_). ^31^P–NMR: δ 42.35 (s).

**Synthesis of (Cy_3_P)Au(C≡C–2-[C_14_H_6_-9,10-diethoxy]-7–C≡C)Au(PCy_3_) (5b).** AuCl(PCy_3_) (0.145 g, 0.283 mmol) and Bu*^t^*OK (0.091 g, 0.811 mmol) were added to a solution of compound **4** (0.044 g, 0.140 mmol) in methanol (15 mL) under a N_2_ atmosphere and the mixture was stirred overnight. Reduction in volume of the solvent afforded a solid residue that was dissolved in CH_2_Cl_2_ and precipitated by adding ether, to afford **5b** (0.131 g, 74%) as a yellow powder. HR ESI MS [C_58_H_83_^197^Au_2_O_2_P_2_]: Calcd. 1267.5200, found 1267.5192. Elemental analysis for C_58_H_83_Au_2_O_2_P_2_, Calcd (found): C, 54.98 (55.11) and H, 6.52 (6.22). IR (solid): 2100 ν(C≡C). UV-Vis (CHCl_3_): 327 [1.47], 342 [2.25], and 360 [1.89]. ^1^H–NMR (CDCl_3_): δ 1.46 (t, ^3^*J_HH_* = *7* Hz) [6H, 2 × CH_3_], 1.25–2.10 (m) [66H, 2 × PCy_3_], 4.25 (q, ^3^*J_HH_* = *7* Hz) [4H, 2 × OCH_2_], 7.68 (dd, ^3^*J_HH_* = 9 Hz, ^4^*J_HH_* = 2 Hz) [2H, H_4_], 8.05 (d, ^3^*J_HH_* = 9 Hz) [2H, H_6_], and 8.75 (d, ^4^*J_HH_* = 2 Hz) [2H, H_3_]. ^13^C–NMR (CDCl_3_): 15.94 (2C, 2 × CH_3_), 25.93, 26.95, 27.00, 27.14, 27.20, 30.72, 30.77, 33.15, 33.28 (36C, 2 × PCy_3_), 68.69 (2C, 2 × OCH_2_), 104.36 (2C, C_8_), 121.66, 122.48, 127.97, 128.19, 130.60 (14C, C_2_, C_3_, C_4_, C_5_, C_6_, C_7_), 143.00 (2C, C_1_), and C_9_ is not observed. ^31^P–NMR: δ 56.31 (s).

### 3.4. DNA Binding Studies

#### 3.4.1. Determination of the DNA Binding Constant using UV-Vis Absorption

The concentration of the ct-DNA stock solution in distilled water was determined from the reported molar absorptivity at 260 nm (6600 M^−1^ cm^−1^). The ratio of absorbance at 260 to that at 280 nm (1.8) confirmed that the DNA was free from protein impurities [55]. Spectroscopic studies were conducted by maintaining the concentrations of the compounds at a constant value (20 µM) while varying the concentration of ct-DNA (minimum amount of DMSO was employed to maintain the compounds solubility during the experiment). The spectroscopic responses were tracked after allowing the solution to incubate for 2 min. A pH value of 7.4 was maintained in all the experiments using phosphate buffer. From the absorbance values, the Benesi–Hildebrand equation (Equation (1)) was used to evaluate the binding constants (K_b_) of the compounds with ct-DNA [56].

[A_o_/(A − A_o_)] = [ε_g_/(ε_h-g_ − ε_g_)] + {[ε_g_/(ε_h-g_ − ε_g_)] × [1/(K_b_ [DNA])} (1)

A_o_/A − A_o_ was plotted against 1/[DNA], and the K_b_ values were calculated from the ratio of the intercept to the slope [56] (A_o_ and A are the absorbance values of the compounds in the absence and presence of ct-DNA, respectively). 

#### 3.4.2. Determining the Mode of Interaction by Fluorescence

Solutions of **4**, **5a**, and **5b** of the same concentration (1.00 × 10^−5^ M) in the minimum amount of DMSO in buffer solutions were treated with varying amounts of ct-DNA solution in buffer, and the emission spectra were monitored [57].

### 3.5. Molecular Docking Studies

Molecular Operating Environment (MOE) 2008.10 (Chemical Computing Group Inc., Quebec, Canada, 2008) was used to perform the molecular docking studies. A Gaussian contact surface was drawn around the binding sites enclosing the van der Waals surface. Docking studies were undertaken to assess the binding free energy of the complexes inside the DNA. The docking scores were first acquired utilizing the London dG scoring function in the MOE software, and then were improved using two unrelated refinement methods. The Grid-Min pose and Force-Field were employed to confirm that the refined poses of the complexes were geometrically correct. Bond rotations were allowed, and the best five binding poses were then examined. The docking poses of the ethidium bromide, **4**, **5a**, **5b** and the co-crystallized structure of the B-DNA were docked (RSCP PDB code: 1BNA). RMSD values were used to assess the best binding pose.

### 3.6. Anticancer Activity and Cytotoxcicity

The cells were provided by the Egyptian Holding Company for Biological Products and Vaccines (VACSERA), Giza, Egypt, and were maintain in a tissue culture unit. The growth of the cells was undertaken in a RBMI-1640 medium, sourced with 10% heat inactivated FBS, 50 units/mL of penicillin, and 50 mg/mL of streptomycin, and reserved in a humidified atmosphere containing 5% CO_2_ [58,59]. The cells were kept as a monolayer culture by serial subculturing. Cell culture reagents were obtained from Lonza (Basel, Switzerland). The assessment of the anticancer activity of the compounds was obtained against the MCF-7 cell line (breast cancer), HEPG-2 cell line (liver cancer), PC-3 cell line (prostate cancer), and MOLT-4 cells (leukemia).

The sulforhodamine B (SRB) was used to determine cytotoxicity using the assay method, as previously described by Skehan et al. [60]. Collections of cells were subcultured using 0.25% trypsin-EDTA, and then seeded in 96-well plates at 1000 to 2000 cells/well in a RBMI-1640-supplemented medium. After 24 h, the cells were incubated for 72 h at five different concentrations of the synthesized compounds (10^−4^, 10^−5^, 10^−6^, 10^−7^, 10^−8^ M) in DMSO. After 72 h treatments, the cells were fixed with 10% trichloroacetic acid for 1 h at 4 °C. Wells were stained for 10 min at room temperature with 0.4% SRB (sulforhodamine B) dissolved in 1% acetic acid. The plates were subjected to air drying for 24 h, and the dye was solubilized with Tris hydrochloride for 5 min using a shaker at 1600 rpm. The OD (optical density) of each well was measured spectrophotometrically at 564 nm with an ELISA microplate reader (ChroMate 4300, Awareness Technology, FL, USA). Calculations of IC50 values were obtained from a Boltzmann sigmoidal equation for the response as a function of the concentration, using nonlinear regression fitting models (Graph Pad, Prism Version 5).

## 4. Conclusions

In this article, we have described the synthesis of 3,6-diethynyl-9,10-diethoxyphenanthrene from phenanthrene together with two binuclear gold(I) complexes obtained via metal alkynyl bond-formation reactions. Spectroscopic studies (absorption and emission) were carried out for the metal complexes and their acetylene ligand, showing a red shift in the absorption maxima and emission wavelength of the ligand upon complexation. The calculated K_b_ values of the compounds showed that the 3,6-diethynyl-9,10-diethoxyphenanthrene (**4**) has better binding affinity toward ct-DNA than ethidium bromide. Functionalizing the phenanthrenyl ligand with AuPPh_3_ enhances the binding constant of the phenanthrenyl ligand while AuPCy_3_ slightly diminishes the binding constant, an observation which can be attributed to the increased steric hindrance associated with the latter. According to the docking studies, **4** establishes pi–pi interactions with several deoxyadenosine (DA) and thymidine (DT) nucleotides in the two strands of the ct-DNA, in addition to donor–acceptor interactions of the acetylene and the deoxyadenosine (DA) nucleotides. In contrast, the gold complexes interact with nucleotides from one strand of the DNA, forming several pi–pi interactions. The results of the molecular docking studies show good agreement with the experimental findings of the DNA-binding study. The anticancer screening of the gold complexes against four cell lines showed remarkable and promising cytotoxicity as compared with that of cisplatin.

In conclusion, a strongly intercalating phenanthrenyl system has been synthesized which has the potential to be decorated with diverse metal centers through metal-alkynyl bond formation. The functionalization of the phenanthrenyl system with the gold centers has introduced promising anticancer activities while maintaining the DNA binding capabilities. The two components (phenanthrenyl and metal centers) of our complexes can be altered independently. To improve the binding (targeting) of the complex, the phenanthrenyl-based ligand can be modified while the anticancer properties (including the physicochemical properties) can be fine-tuned by modifying the metal centers and the co-ligands. 

## Figures and Tables

**Table 1:** DNA binding affinities toward ct-DNA determined via different methods.

Compound	K_b_ (DNA Binding Constant)
Ethidium Bromide	5.00 × 10^5^
**4**	7.40 × 10^5^
**5a**	8.71 × 10^5^
**5b**	7.00 × 10^5^

**Table 2:** Docking study results for **4**, **5a**, and **5b**.

Compound	Docking Score	Ligand	Receptor	Interaction	Distance
Ethidium Bromide	−4.6372	Phenanthridine	5-ring DA 18 (B)	pi–pi	3.80
		Phenanthridine	6-ring DT 19 (B)	pi–pi	3.83
**4**	−5.1630	C≡C	OP2 DA 17 (B)	H-donor	3.11
		Phenanthrene	6-ring DA 17 (B)	pi–pi	3.87
		Phenanthrene	5-ring DA 17 (B)	pi–pi	3.56
		Phenanthrene	6-ring DT 19 (B)	pi–pi	3.36
		Phenanthrene	6-ring DA 18 (B)	pi–pi	3.21
		Phenanthrene	6-ring DA 6 (A)	pi–pi	3.95
**5a**	−5.4070	Phenanthrene	5-ring DA 17 (B)	pi–pi	3.28
		Phenanthrene	5-ring DA 18 (B)	pi–pi	3.77
		Phenanthrene	5-ring DA 17 (B)	pi–p	3.65
		Phenanthrene	6-ring DA 18 (B)	pi–pi	3.63
		Phenanthrene	6-ring DA 17 (B)	pi–pi	3.09
		Phenanthrene	5-ring DA 17 (B)	pi–pi	3.75
		Phenanthrene	6-ring DT 19 (B)	pi–pi	3.99
**5b**	−4.1090	Phenanthrene	5-ring DA 18 (B)	pi–pi	3.41
		Phenanthrene	6-ring DT 19 (B)	pi–pi	3.50

**Table 3:** Anticancer activities of selected compounds in DMSO solutions.

Complex	IC50 ± SD (μM)			
	MCF-7	HEPG-2	PC-3	MOLT-4
**3**	193.37 ± 0.20	164.56 ± 0.20	149.28 ± 0.22	177.87 ± 0.16
**4**	283.48 ± 0.13	245.78 ± 0.15	227.63 ± 0.06	269.02 ± 0.10
**5a**	22.58 ± 0.03	26.01 ± 0.03	27.46 ± 0.01	25.91 ± 0.03
**5b**	18.63 ± 0.03	27.94 ± 0.03	27.31 ± 0.01	20.28 ± 0.03
Cisplatin	16.00 ± 0.06	-	39.99 ± 0.05	-

## Supplementary Materials

The following are available online, Figure S1: HR Mass spectrometry of Compound 1, Figure S2: IR of Compound 1, Figure S3: H–NMR of Compound 1, Figure S4: CNMR of Compound 1, Figure S5: HR Mass spectrometry of Compound 2, Figure S6: HR Mass spectrometry of Compound 2, Figure S7: HR Mass spectrometry of Compound 2, Figure S8: IR of Compound 2, Figure S9: HNMR of Compound 2, Figure S10: CNMR of Compound 2, Figure S11: HR Mass spectrometry of Compound 3, Figure S12: IR of Compound 3, Figure S13: HNMR of Compound 3, Figure S14: CNMR of Compound 3, Figure S15: HR Mass spectrometry of Compound 4, Figure S16: IR of Compound 4, Figure S17: HNMR of Compound 4, Figure S18: CNMR of Compound 4, Figure S19: HR Mass spectrometry of Compound 5a, Figure S20: IR of Compound 5a, Figure S21: HNMR of Compound 5a, Figure S22: CNMR of Compound 5a, Figure S23: PNMR of Compound 5a, Figure S25: HNMR of Compound 5b, Figure S24: HR Mass spectrometry of Compound 5b, Figure S26: CNMR of Compound 5b, Figure S27: PNMR of Compound 5b, Figure S28: HNMR of 3,6-dibromophenanthrerne-9,10-dione, Figure S29: CNMR of 3,6-dibromophenanthrerne-9,10-dione.

## Abbreviations

TG-CA	Thymine/guanine-cytosine/adenine.
Ct-DNA	calf-thymus DNA
MCF-7	abbreviated from Michigan Cancer Foundation-7; a breast cancer cell line.
HEPG-2	hepatic liver carcinoma cell line.
PC-3	hhuman prostate cancer cell line.
MOLT-4	Human acute lymphoblastic leukemia cell line.
