# Supplementary material for: DNA-Binding and Anticancer Activity of Binuclear Gold(I) Alkynyl Complexes with a Phenanthrenyl Bridging Ligand
Source: Molecules. 2020 Feb 25;25(5):1033. doi: 10.3390/molecules25051033 (PMC7179095; doi:10.3390/molecules25051033)
Supplement: Supplementary file 1 [file molecules-25-01033-s001.pdf]

DNA-Binding and Anticancer Activity of Binuclear Gold(I) Alkynyl Complexes with Phenanthrenyl Bridging Ligands

Mona S. Alsaeedi, Bandar A. Babgi, Mostafa A. Hussein, Magda H. Abdellattif, Mark G. Humphrey

Supporting Information

Figure S 1: HR Mass spectrometry of Compound 1.
Figure S 2: IR of Compound 1.
Figure S 3: HNMR of Compound 1.
Figure S 4: CNMR of Compound 1.
Figure S 5 : HR Mass spectrometry of Compound 2. 4
Figure S 6: HR Mass spectrometry of Compound 2. 4
Figure S 7: HR Mass spectrometry of Compound 2 5
Figure S 8: IR of Compound 2. 5
Figure S 9: HNMR of Compound 2. 6
Figure S 10: CNMR of Compound 2. 6
Figure S 11: HR Mass spectrometry of Compound 3. 7
Figure S 12: IR of Compound 3. 7
Figure S 13: HNMR of Compound 3. 8
Figure S 14: CNMR of Compound 3. 8
Figure S 15: HR Mass spectrometry of Compound 4. 9
Figure S 16: IR of Compound 4. 9
Figure S 17: HNMR of Compound 4. 10
Figure S 18: CNMR of Compound 4. 10
Figure S 19: HR Mass spectrometry of compound 5a. 11
Figure S 20: IR of compound 5a. 11
Figure S 21: HNMR of Compound 5a. 12
Figure S 22: CNMR of Compound 5a. 12
Figure S 23: PNMR of compound 5a. 13
Figure S 25: HNMR of Compound 5b. 14
Figure S 24: HR Mass spectrometry of Compound 5b. 14
Figure S 26: CNMR of Compound 5b. 15
Figure S 27: PNMR of Compound 5b. 15
Figure S 28: HNMR of 3,6-dibromophenanthrerne-9,10-dione. 16
Figure S 29: CNMR of 3,6-dibromophenanthrerne-9,10-dione 16

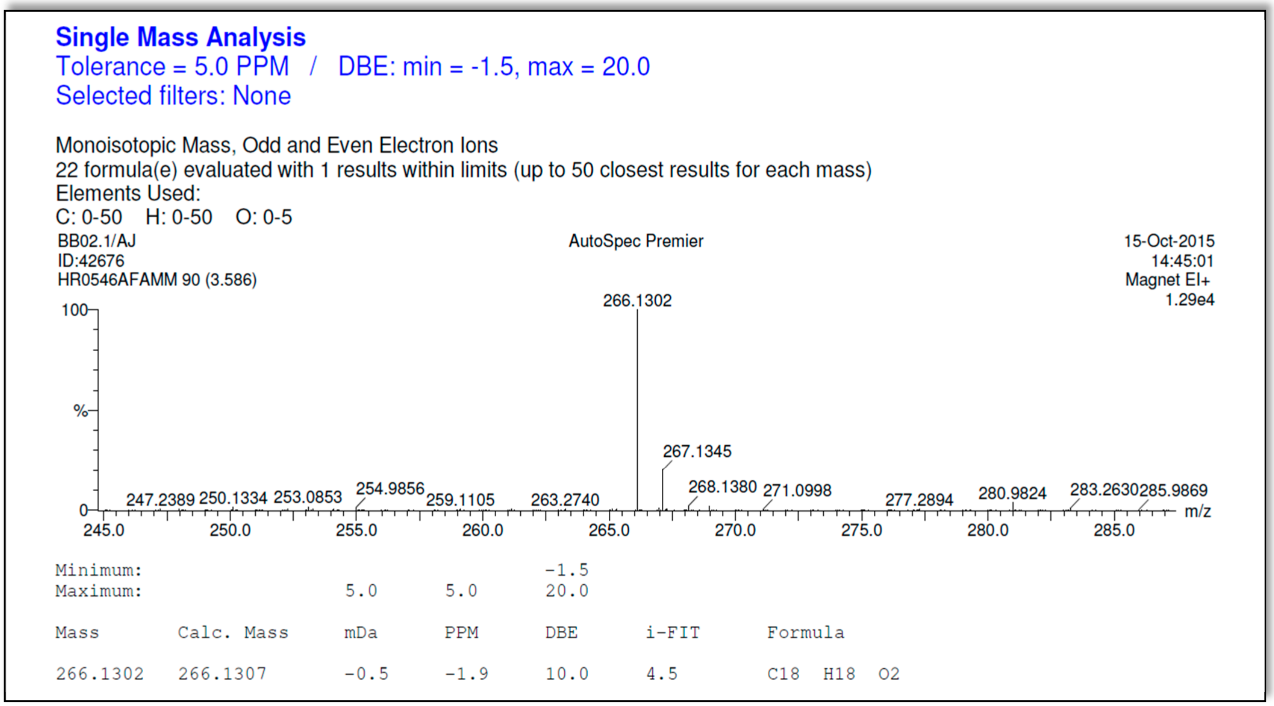

Figure S2. HR Mass spectrometry of Compound 1.

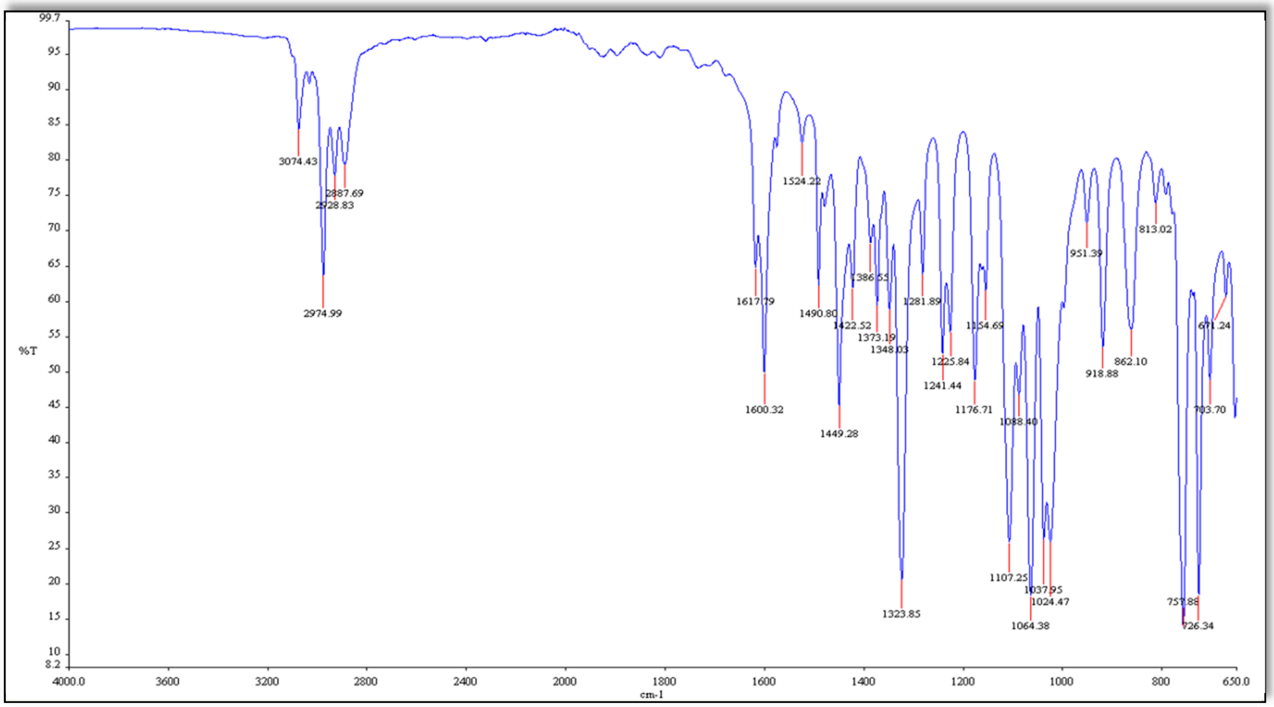

Figure S1. IR of Compound 1.

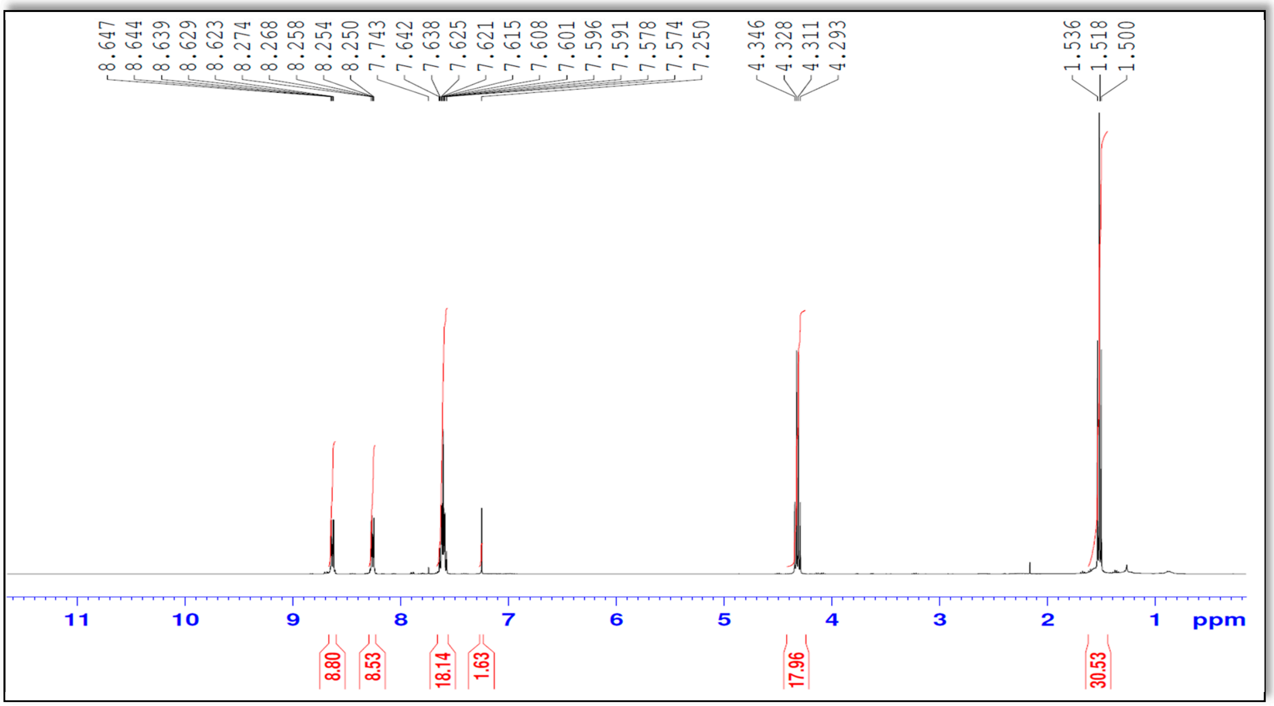

Figure S3. HNMR of Compound 1.

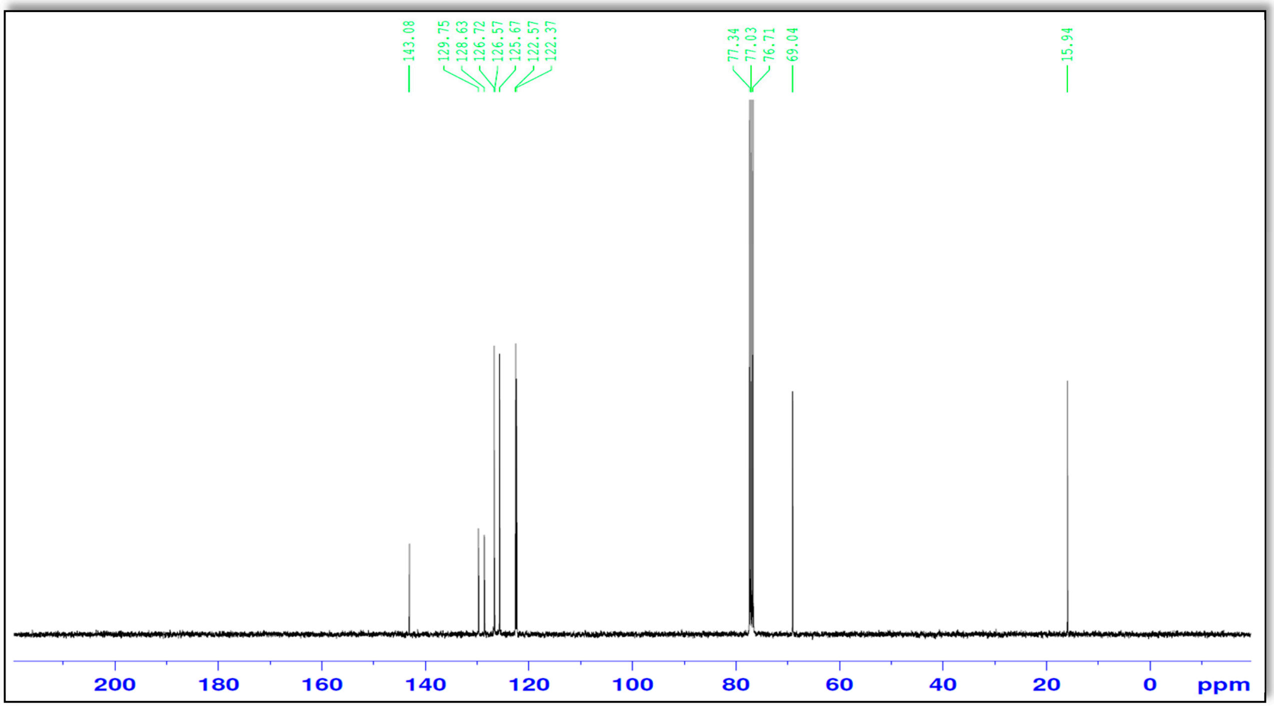

Figure S4. CNMR of Compound 1.

Single Mass Analysis
Tolerance = 5.0 PPM / DBE: min = -1.5, max = 10.0
Selected filters: None

Monoisotopic Mass, Odd and Even Electron Ions
178 formula(e) evaluated with 1 results within limits (up to 50 closest results for each mass)
Elements Used:
C: 0-50 H: 0-50 O: 0-5 79Br: 0-2 81Br: 0-2

BB02.2/AJ AutoSpec Premier 15-Oct-2015
ID:42677 15:15:39
HR0547AFAMM 54 (2.152) Magnet EI+
1.61e3

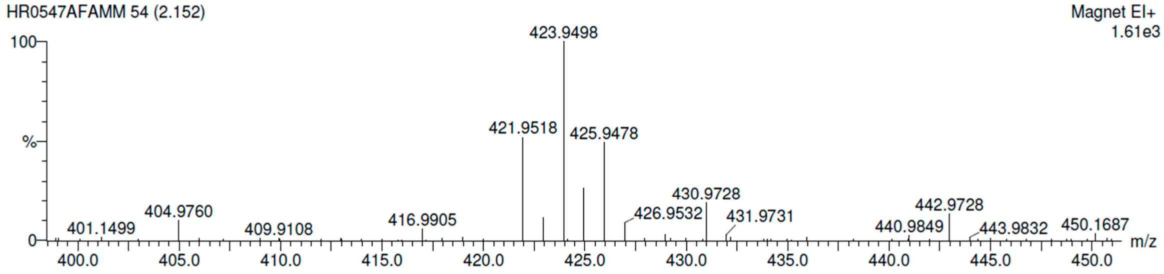

Minimum:				-1.5		
Maximum:		5.0	5.0	10.0		
Mass	Calc. Mass	mDa	PPM	DBE	i-FIT	Formula
421.9518	421.9517	0.1	0.2	10.0	0.5	C18 H16 O2 79Br2

Figure S5. HR Mass spectrometry of Compound 2.

Single Mass Analysis
Tolerance = 5.0 PPM / DBE: min = -1.5, max = 10.0
Selected filters: None

Monoisotopic Mass, Odd and Even Electron Ions
179 formula(e) evaluated with 1 results within limits (up to 50 closest results for each mass)
Elements Used:
C: 0-50 H: 0-50 O: 0-5 79Br: 0-2 81Br: 0-2

BB02.2/AJ AutoSpec Premier 15-Oct-2015
ID:42677 15:15:39
HR0547AFAMM 54 (2.152) Magnet EI+
1.61e3

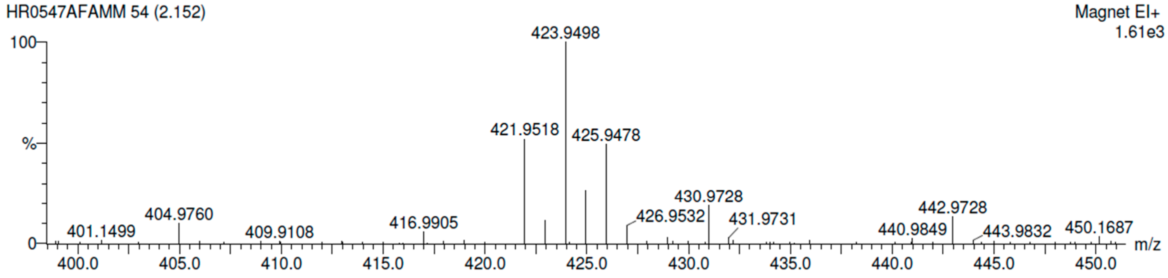

Minimum:				-1.5		
Maximum:		5.0	5.0	10.0		
Mass	Calc. Mass	mDa	PPM	DBE	i-FIT	Formula
423.9498	423.9497	0.1	0.2	10.0	11.1	C18 H16 O2 79Br 81Br

Figure S6. HR Mass spectrometry of Compound 2.

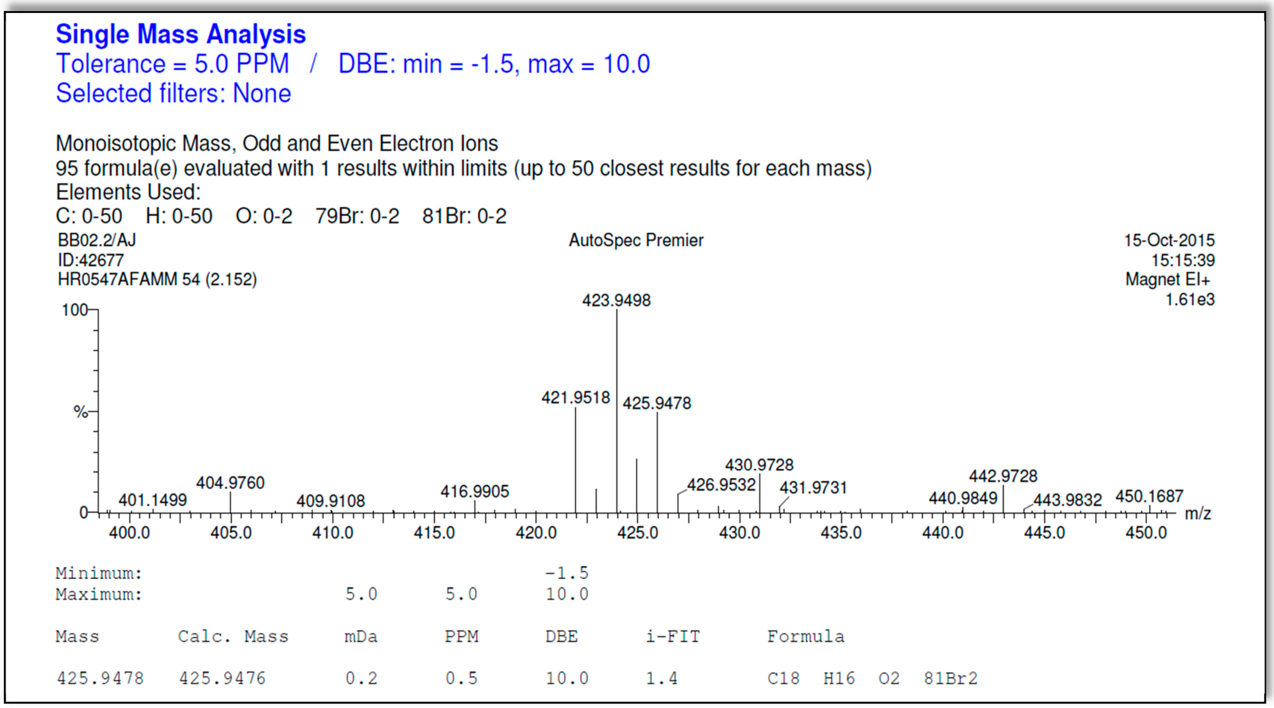

Figure S7. HR Mass spectrometry of Compound 2

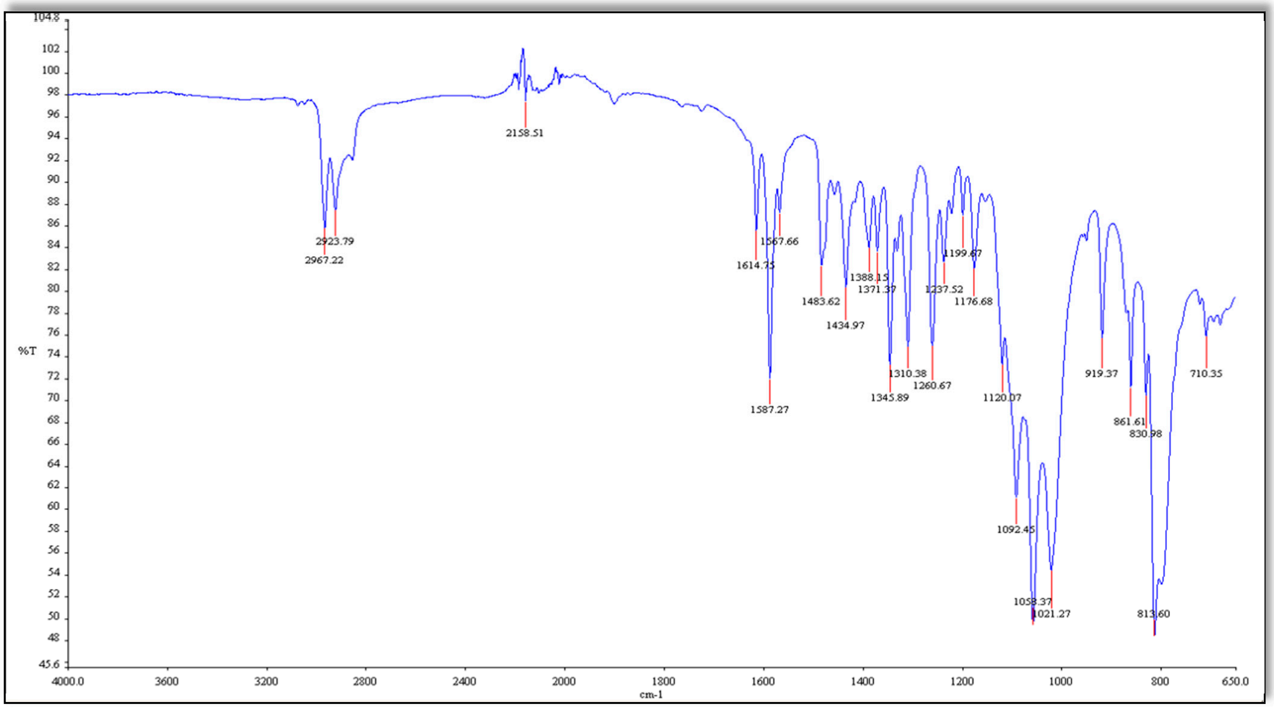

Figure S8. IR of Compound 2.

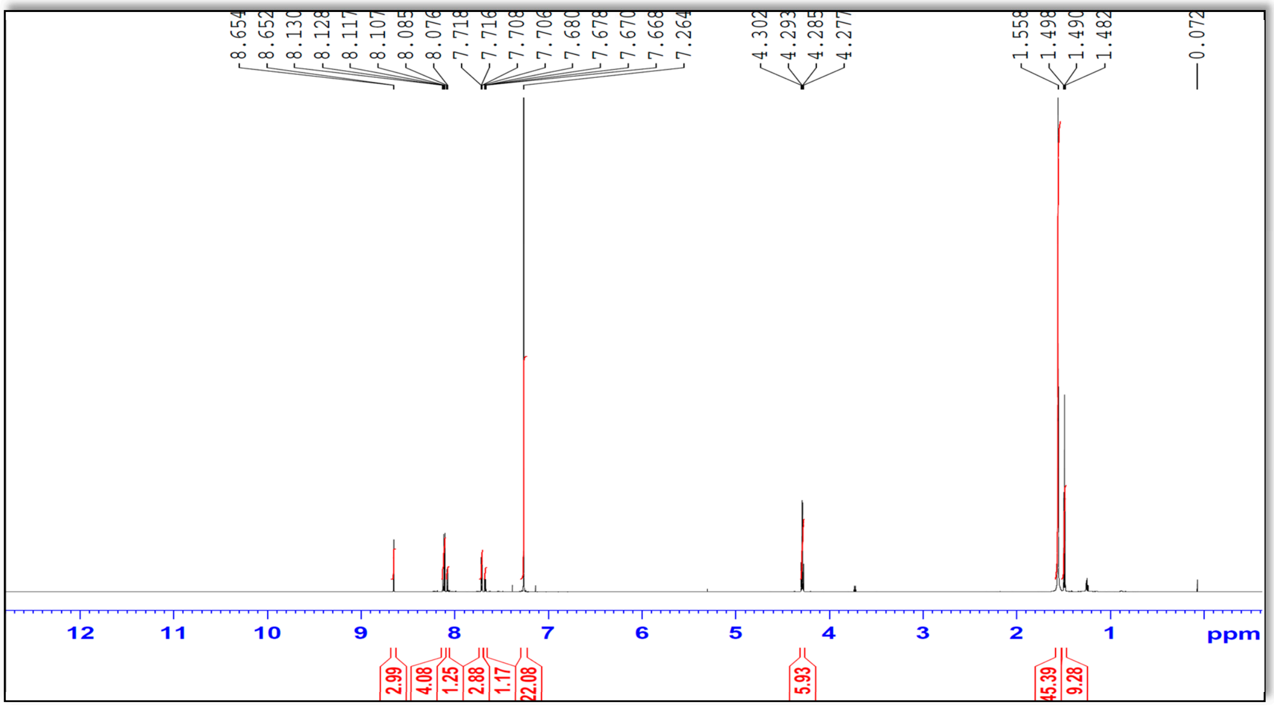

Figure S9. HNMR of Compound 2.

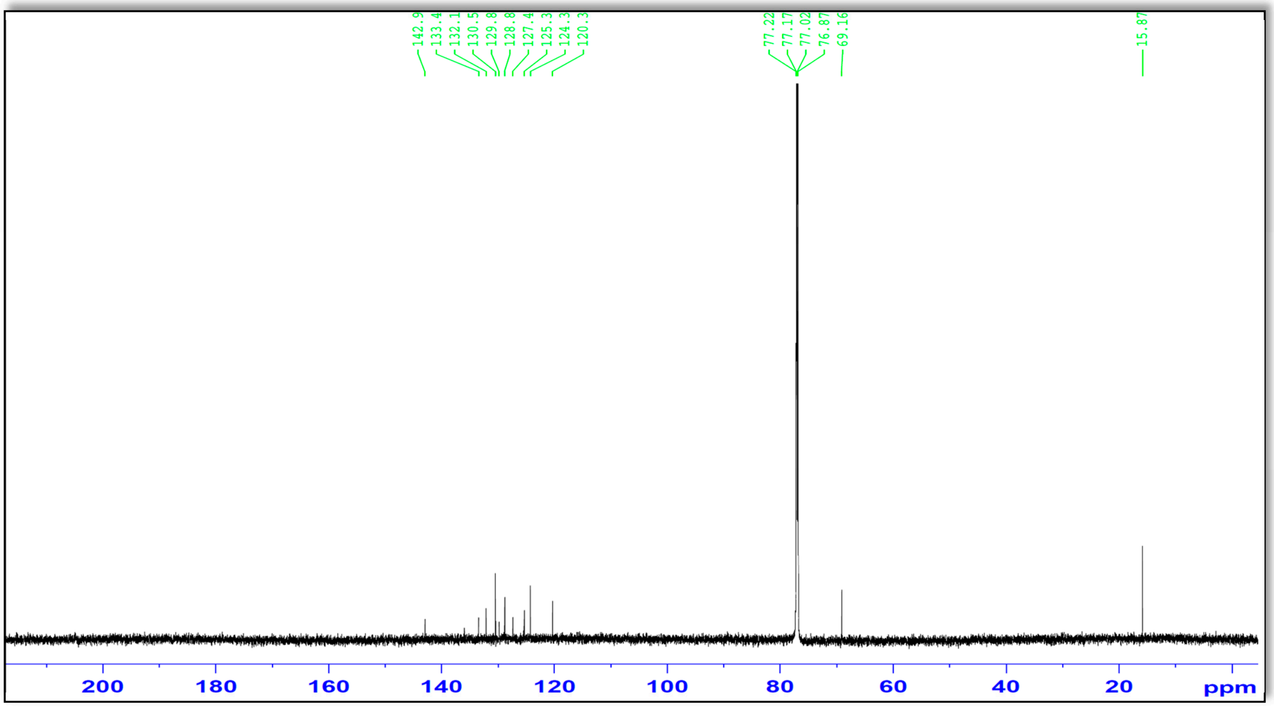

Figure S10. CNMR of Compound 2.

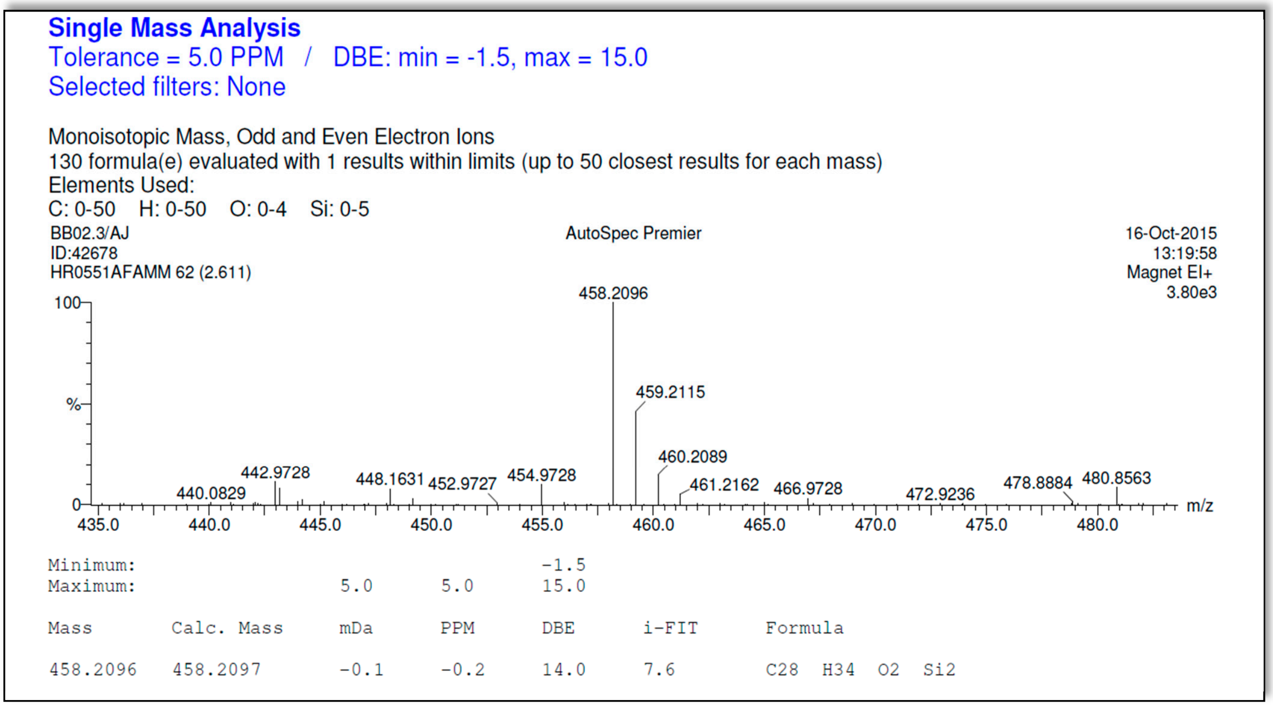

Figure S11. HR Mass spectrometry of Compound 3.

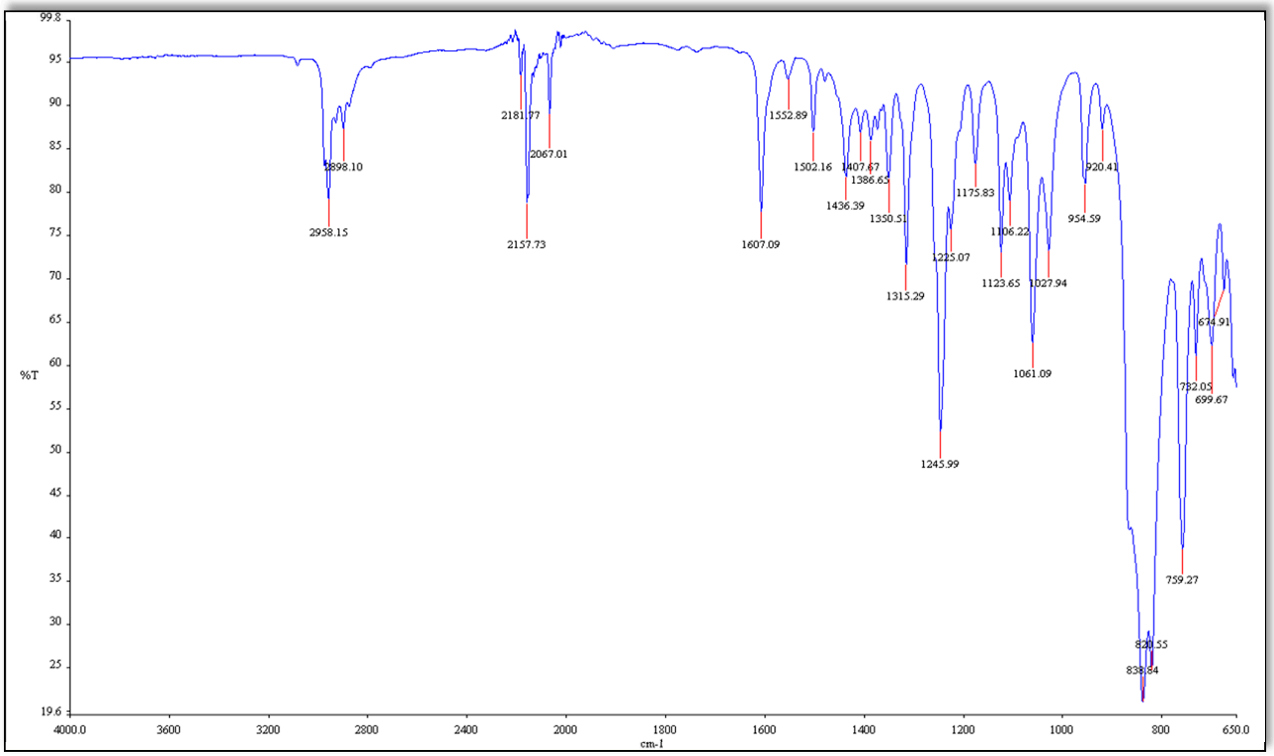

Figure S12. IR of Compound 3.

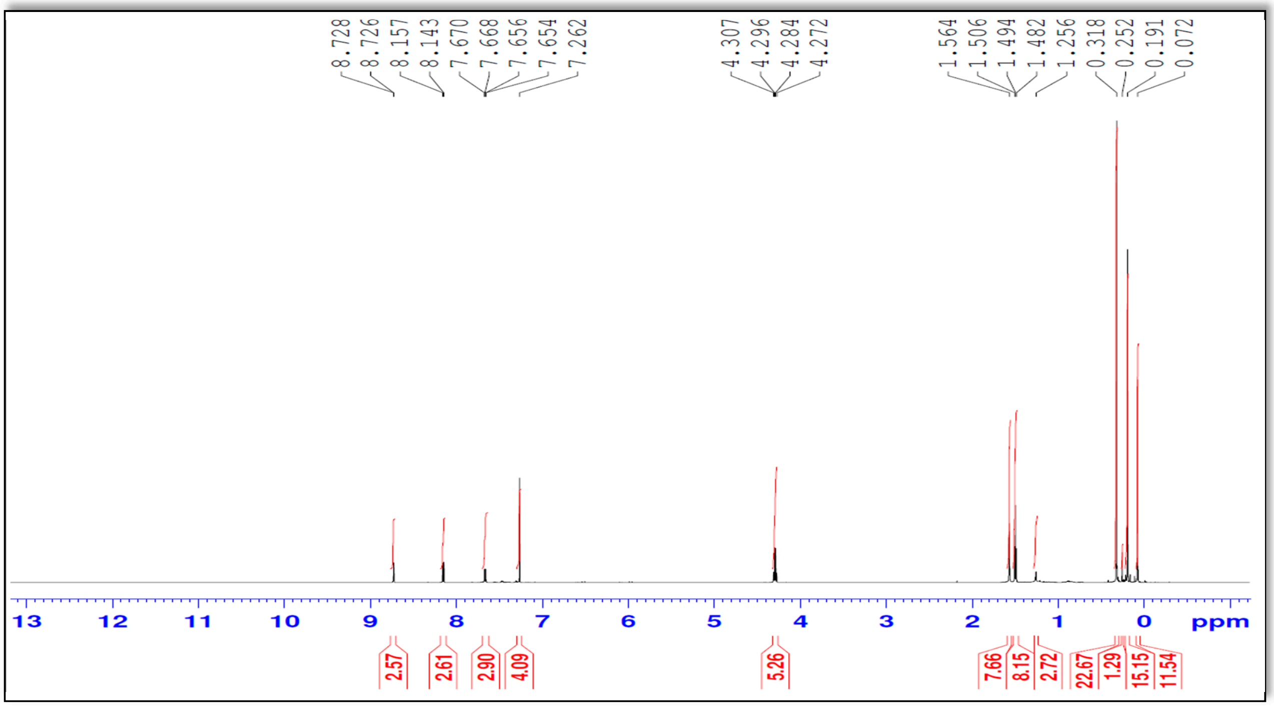

Figure S13. HNMR of Compound 3.

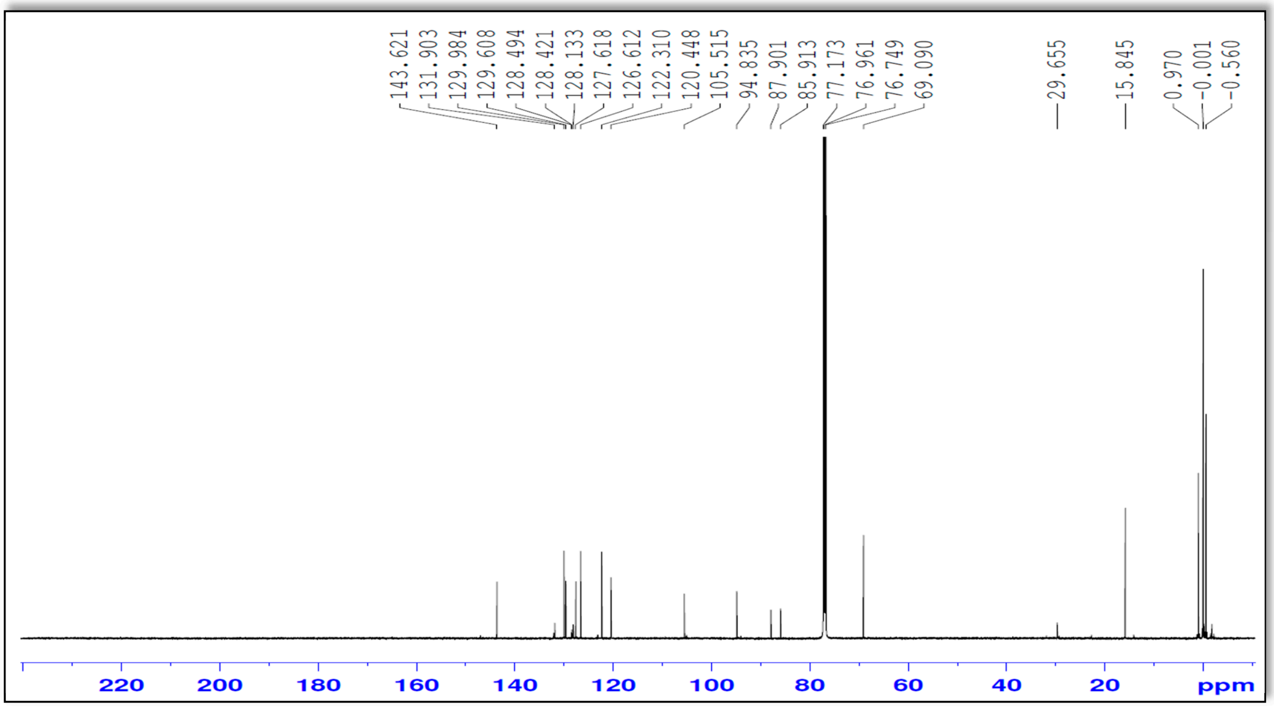

Figure S14. CNMR of Compound 3.

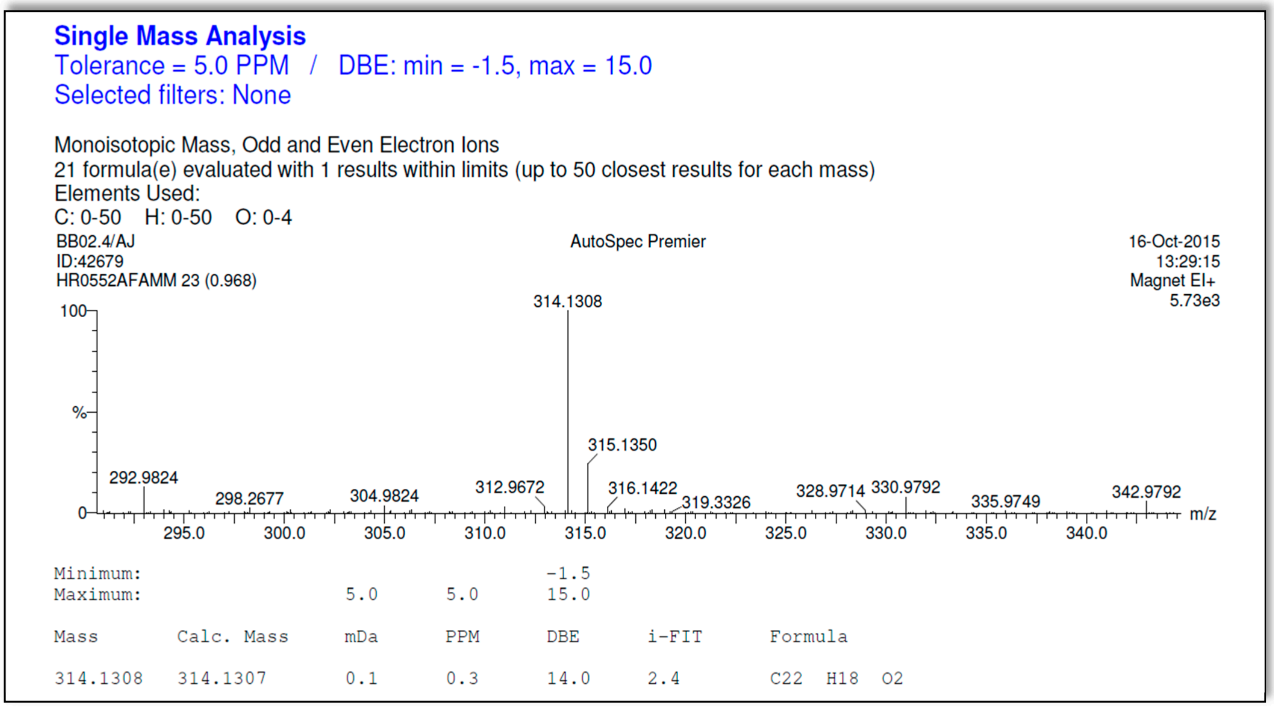

Figure S15. HR Mass spectrometry of Compound 4.

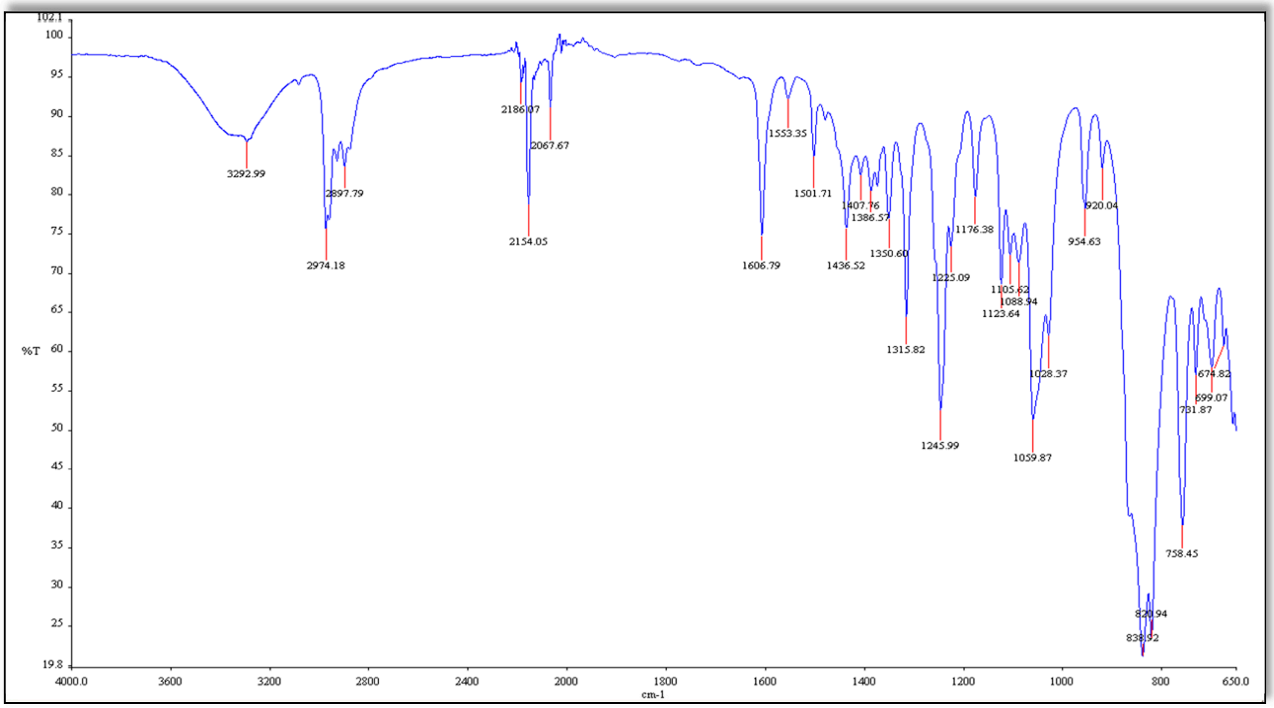

Figure S16. IR of Compound 4.

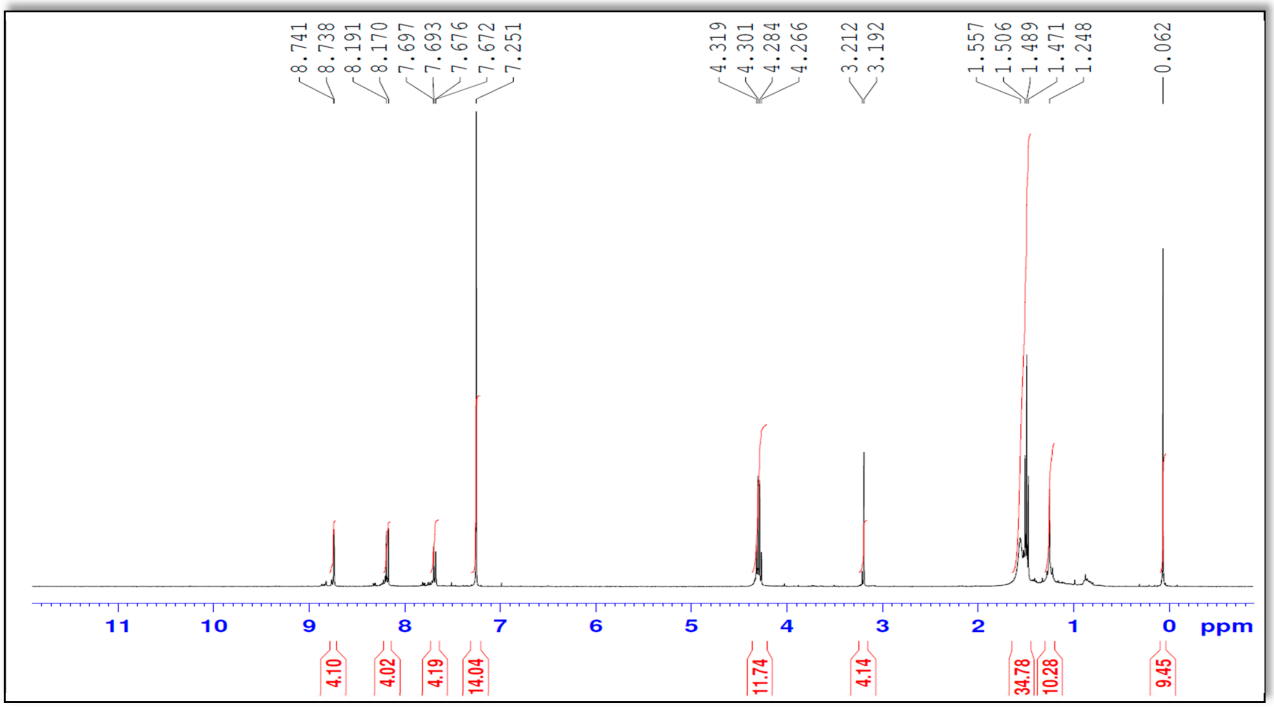

Figure S17. HNMR of Compound 4.

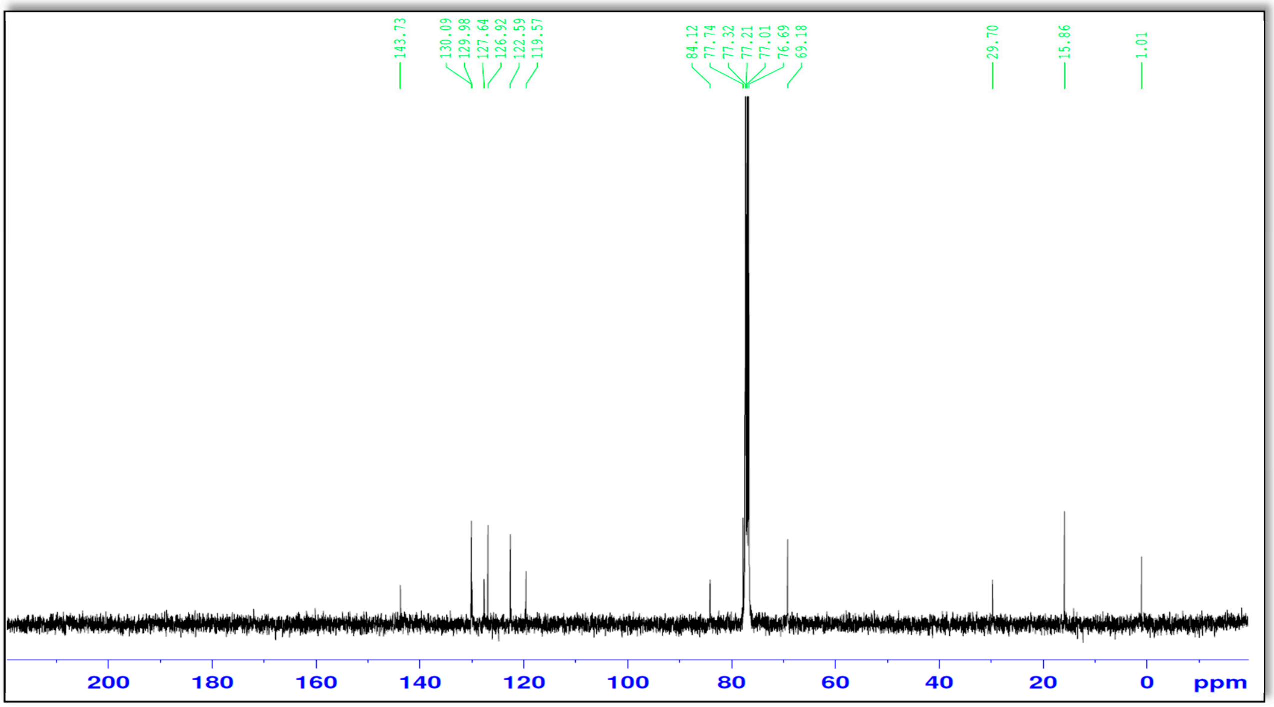

Figure S18. CNMR of Compound 4.

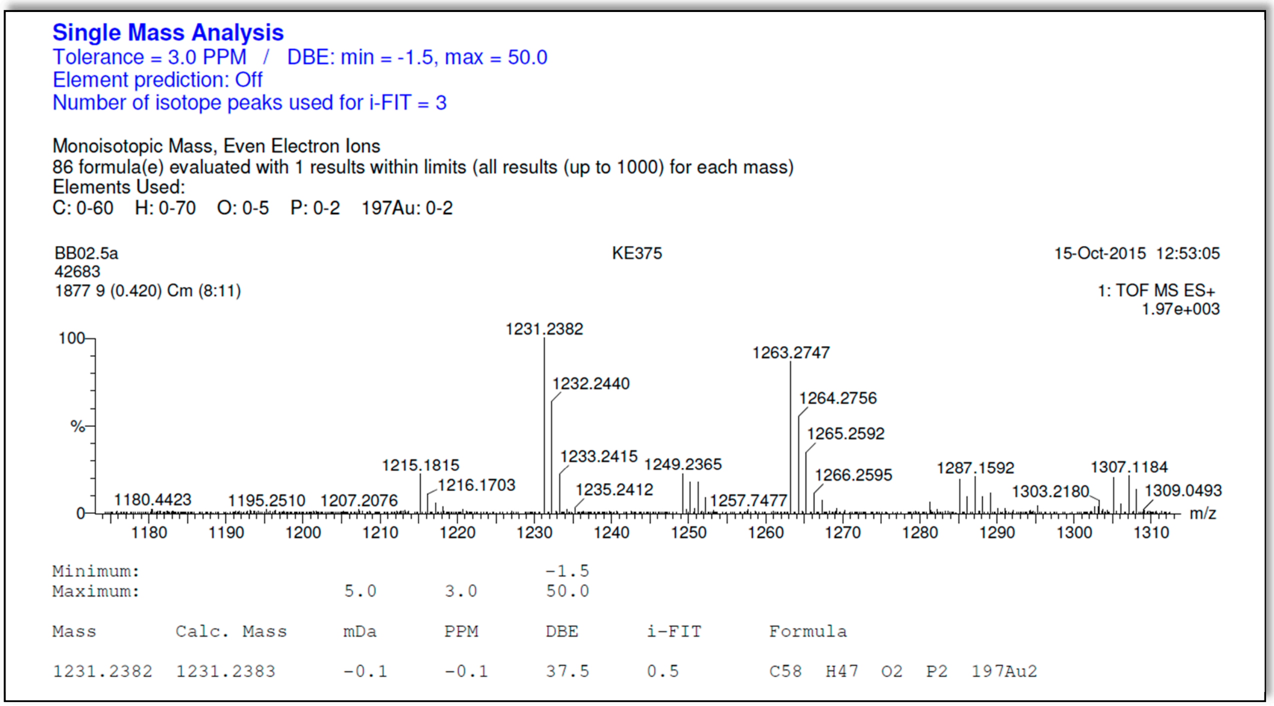

Figure S19. HR Mass spectrometry of compound 5a.

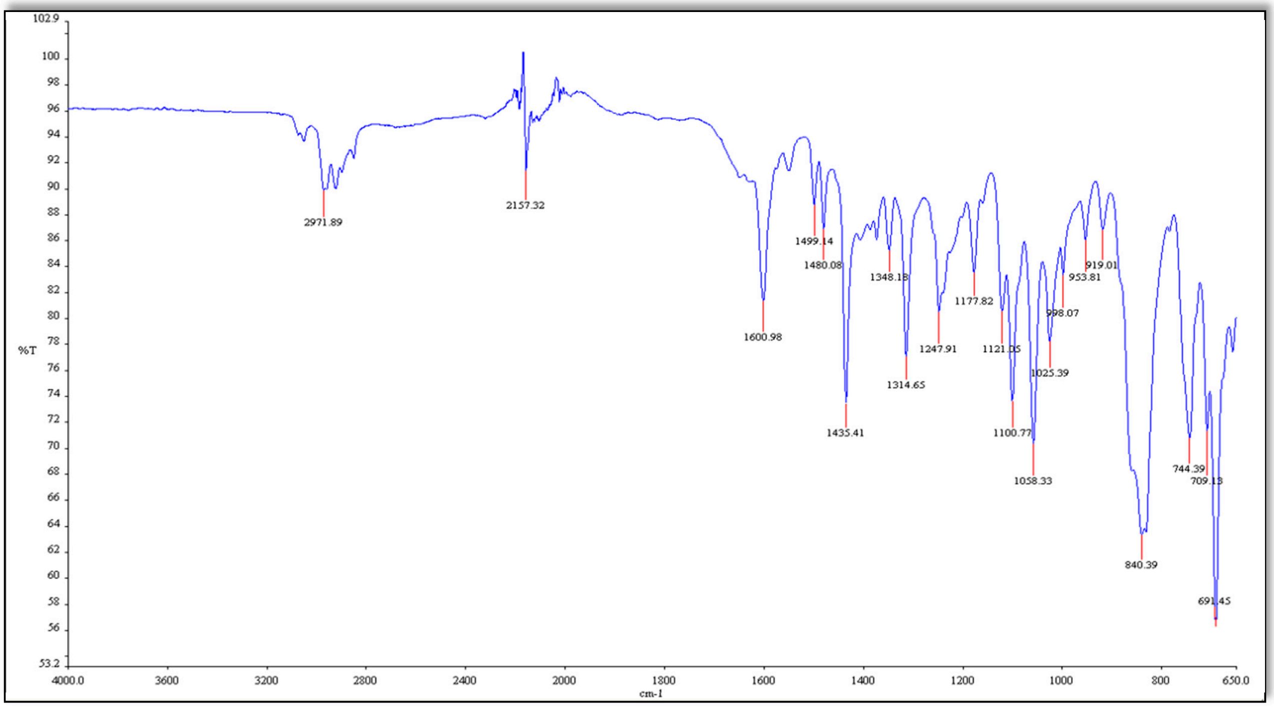

Figure S20. IR of compound 5a.

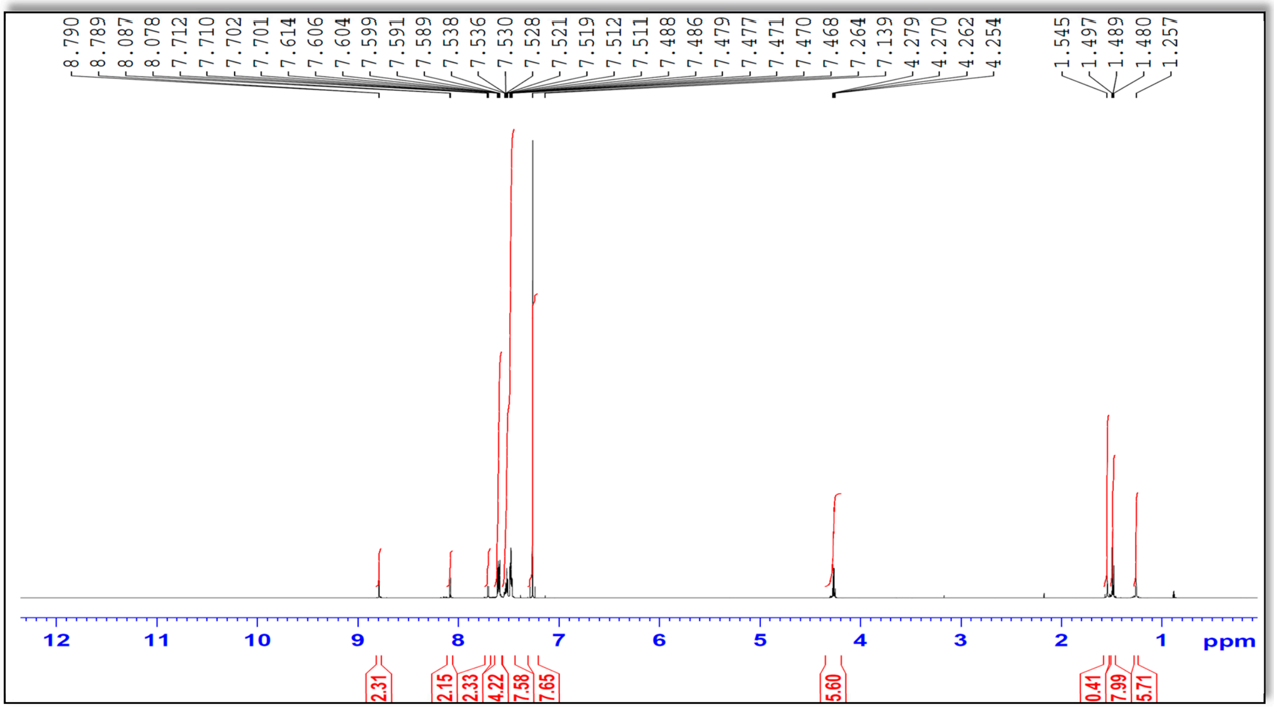

Figure S21. HNMR of Compound 5a.

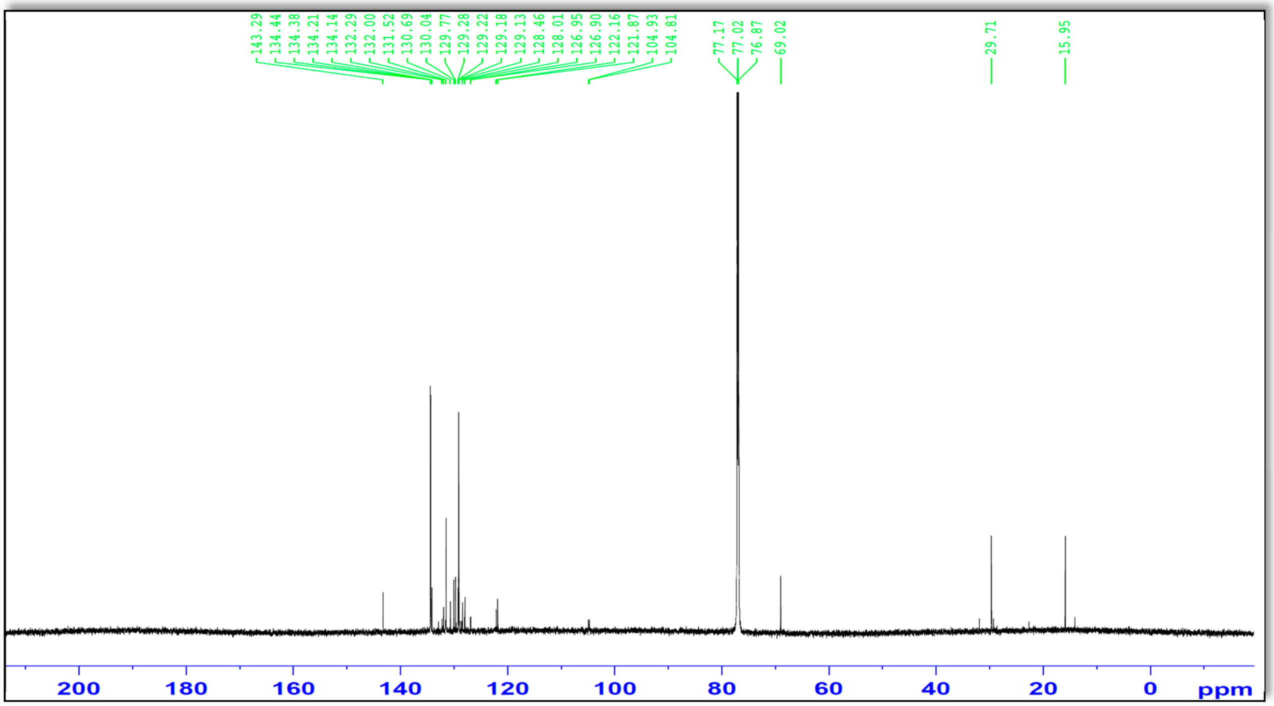

Figure S22. CNMR of Compound 5a.

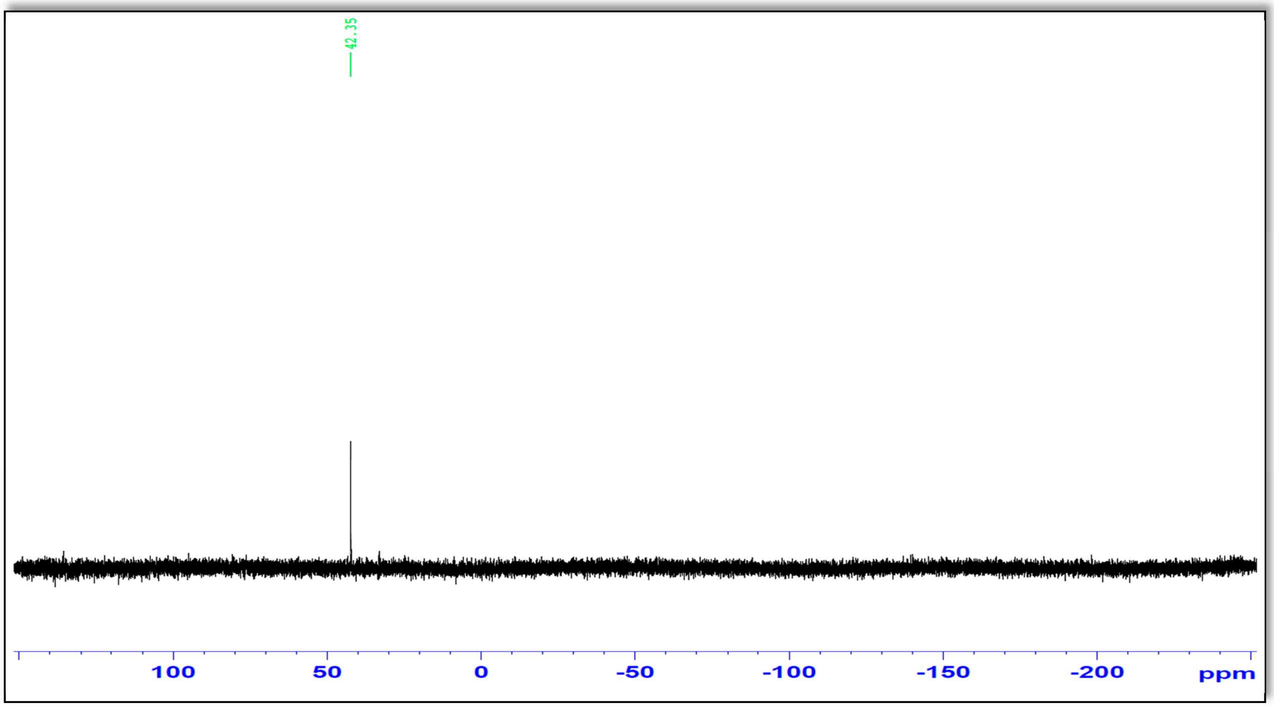

Figure S23. PNMR of compound 5a.

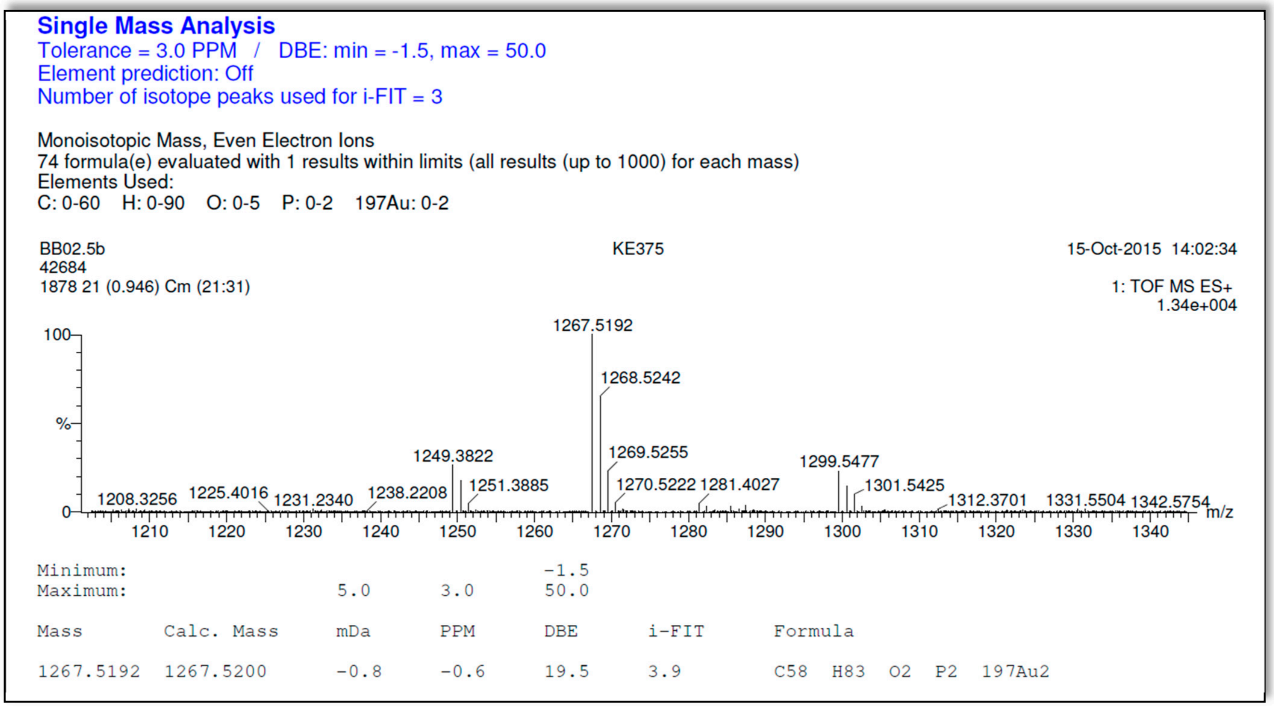

Figure S25. HR Mass spectrometry of Compound 5b.

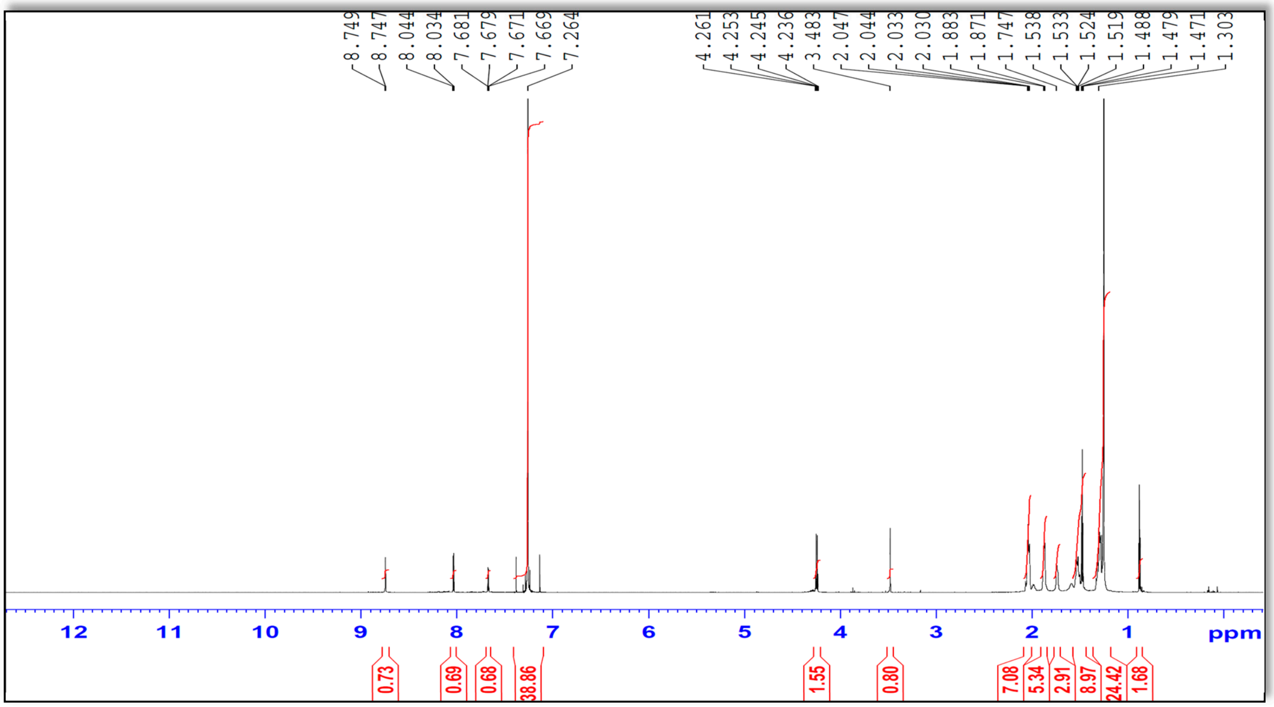

Figure S24. HNMR of Compound 5b.

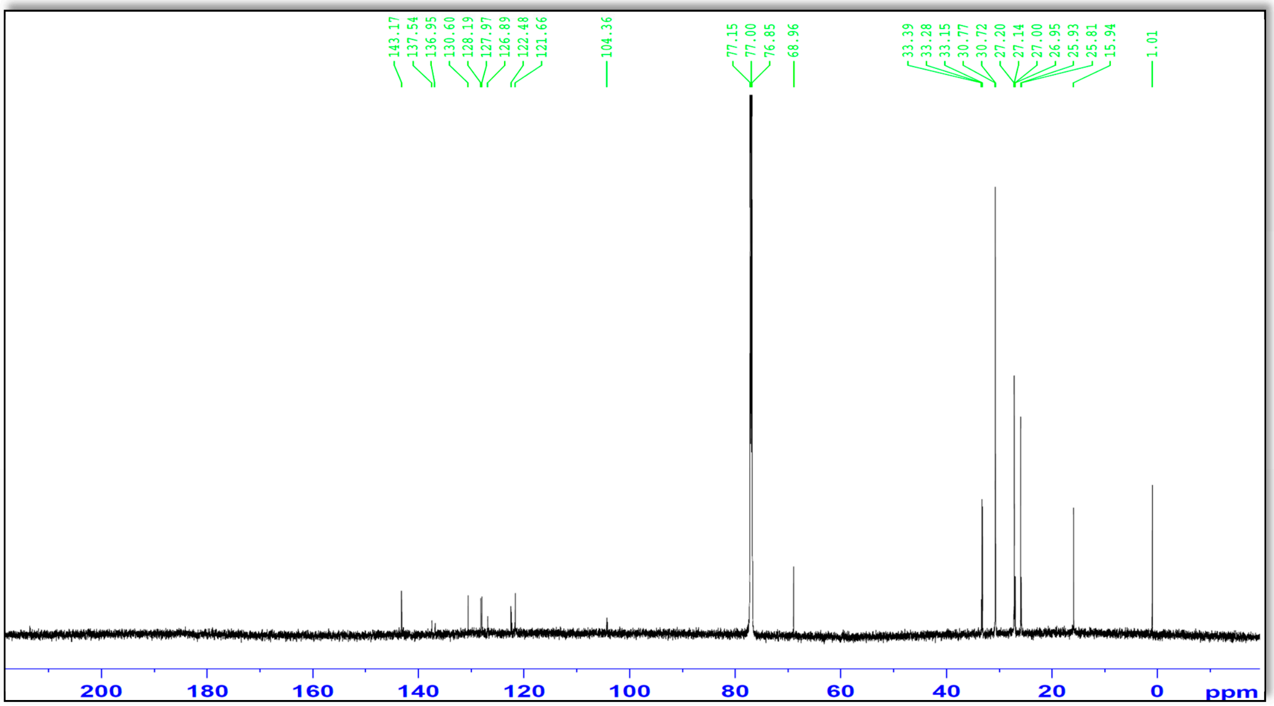

Figure S26. CNMR of Compound 5b.

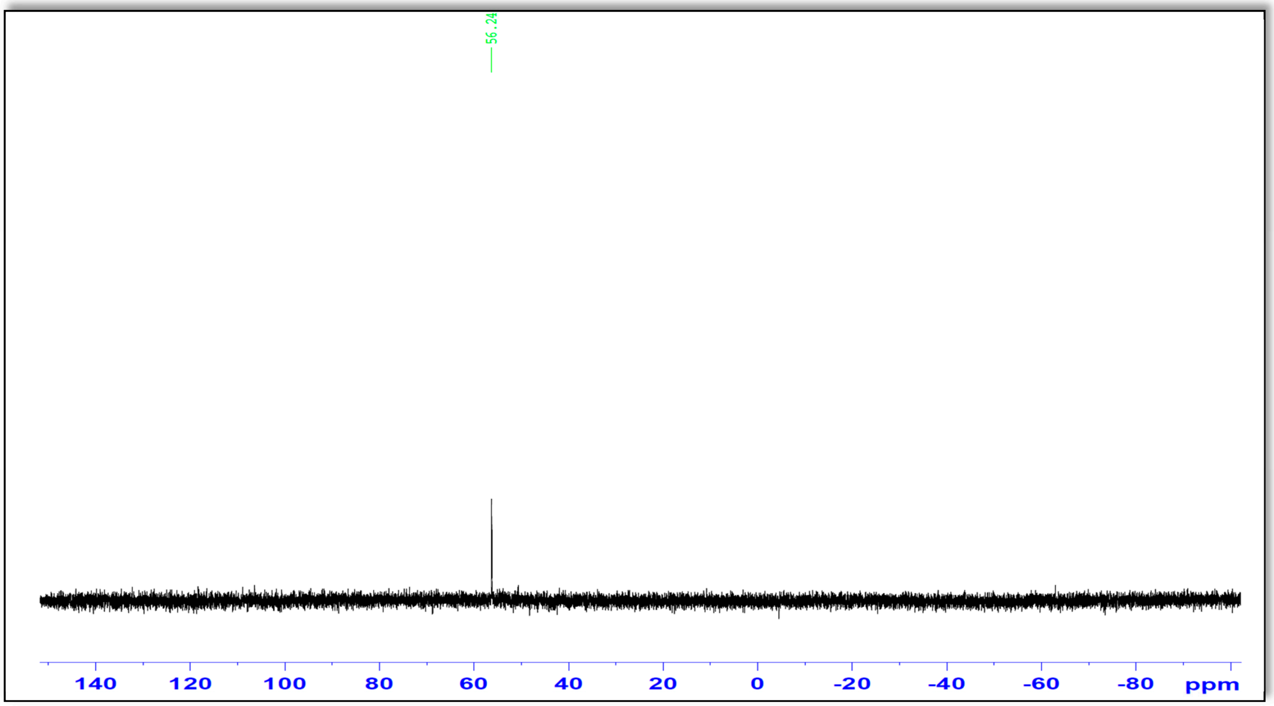

Figure S27. PNMR of Compound 5b.

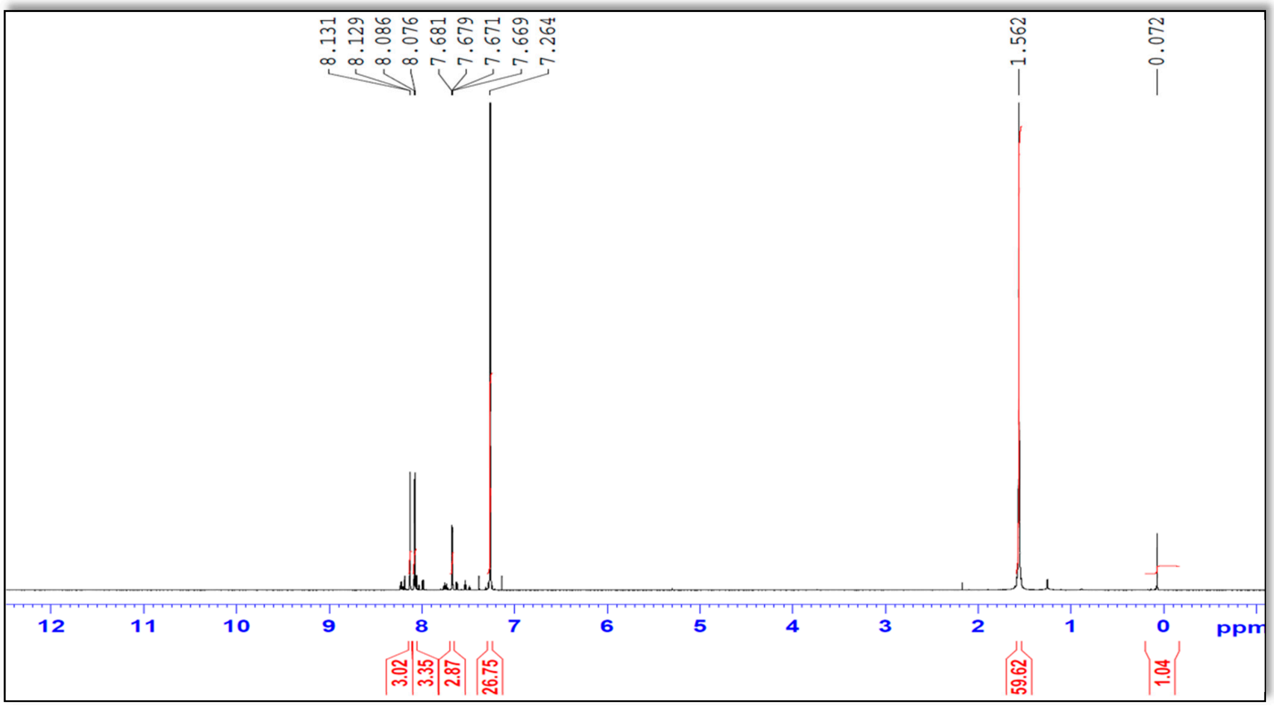

Figure S28. HNMR of 3,6-dibromophenanthrerne-9,10-dione.

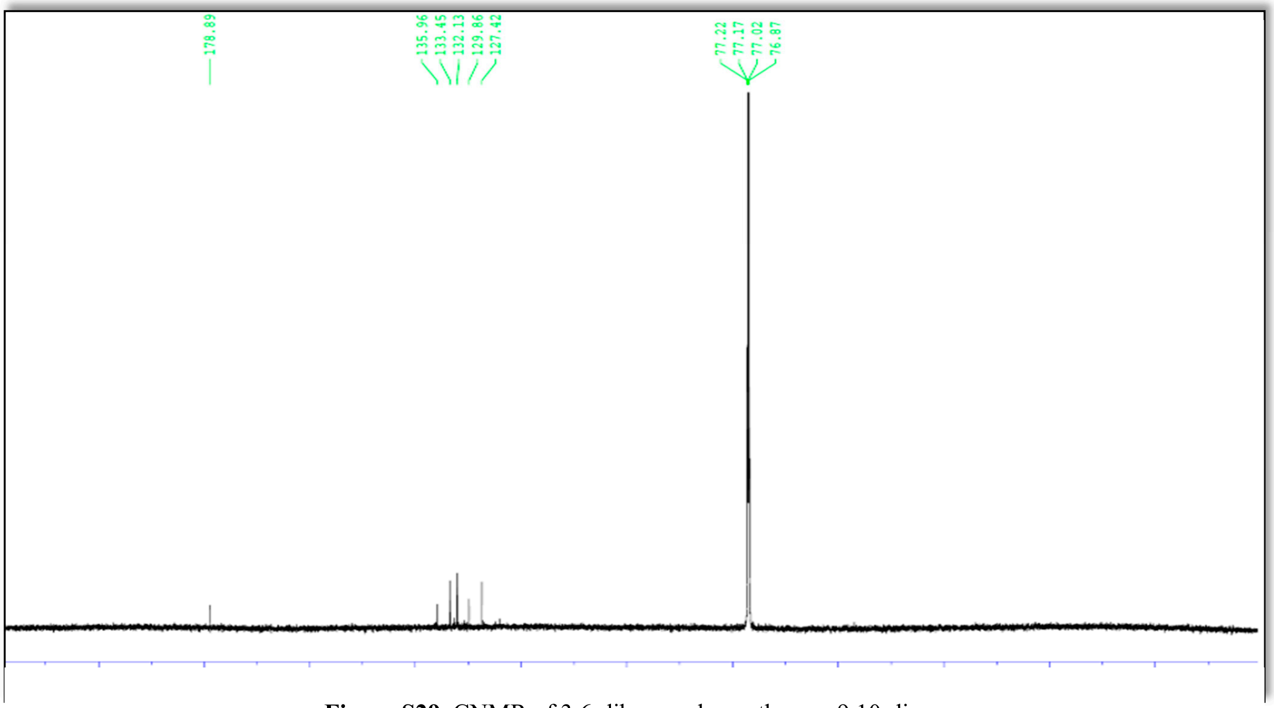

Figure S29. CNMR of 3,6-dibromophenanthrerne-9,10-dione
